# Patients with systemic lupus erythematosus (SLE) have an increased bisphenol A methylation score linked to SLE risk genes and selected clinical subphenotypes

**DOI:** 10.1136/rmdopen-2025-006021

**Published:** 2025-09-25

**Authors:** Holme Vestin, Nina Oparina, Maija-Leena Eloranta, Martina Frodlund, Iva Gunnarsson, Christopher Sjöwall, Elisabet Svenungsson, Lars Rönnblom, Juliana Imgenberg-Kreuz, Dag Leonard

**Affiliations:** 1 Uppsala University Department of Medical Sciences, Uppsala, Sweden; 2 Linkoping University Department of Biomedical and Clinical Sciences, Linköping, Sweden; 3 Division of Rheumatology, Karolinska Institute, Stockholm, Sweden

**Keywords:** Lupus Erythematosus, Systemic, Autoimmunity, Inflammation, Polymorphism, Genetic

## Abstract

**Objectives:**

Bisphenol A (BPA), a xenoestrogen that can alter DNA methylation status, has been implicated in the pathogenesis of systemic lupus erythematosus (SLE). This study aimed to investigate whether methylation changes at BPA-sensitive 5’-C-phosphate-G-3’ (CpG) sites are associated with SLE and clinical subphenotypes.

**Methods:**

A discovery cohort (n=747) and a replication cohort (n=388) including Swedish patients with SLE and healthy controls were investigated using the Illumina HM450k bead chip. BPA-sensitive CpG sites were selected if differentially methylated in ≥2 of 7 BPA exposure studies and supported by cell line data. A BPA_All_ score including 19 CpGs and a BPA_SLE_ score based on three CpG sites co-localised in the genome with SLE risk loci were calculated for each individual, analysed for associations with clinical data and then compared with publicly available transcriptomic data from BPA-treated cells.

**Results:**

Patients with SLE had significantly higher BPA_SLE_ score than controls in the discovery (OR 1.34, p=4.6×10^-13^), replication (OR 1.28, p=1.1×10^-5^) and meta-analysis (OR 1.32, p=3.3×10^-17^). Higher BPA_All_ score was associated with SLE in the discovery cohort (OR 1.05, p=2.3×10^-3^) but not in the replication cohort (OR 1.04, p=0.12) with a significant difference in the meta-analysis (OR 1.05, p=7.0×10^-4^). Both scores were associated with prednisolone treatment (p<0.001), and the BPA_SLE_ score was associated with serositis and autoantibodies (p<0.05). Transcriptomic analysis of BPA-treated cells revealed enrichment in pathways such as interferon and mitogen-activated protein kinase signalling.

**Conclusions:**

Our findings reveal a novel association between BPA exposure and DNA methylation changes in SLE, with potential implications for the regulation of immune-related gene expression.

**WHAT IS ALREADY KNOWN ON THIS TOPIC**

**WHAT THIS STUDY ADDS**

- This study is the first to investigate the potential role of BPA-associated DNA methylation changes in the pathophysiology of systemic lupus erythematosus (SLE), demonstrating that BPA methylation scores are associated with SLE and selected clinical subphenotypes of the disease.
- Data from BPA-treated cells showed expression of genes involved in immune signalling and genes annotated to BPA-sensitive sites.

**HOW THIS STUDY MIGHT AFFECT RESEARCH, PRACTICE OR POLICY**

- BPA may influence SLE susceptibility and disease manifestations through epigenetic mechanisms, supporting the need for precautionary measures to reduce BPA exposure and highlighting the importance of further studies.
- This study establishes a framework for assessing the epigenetic effect of environmental exposures in a disease-specific context, offering a valuable strategy when epigenetic effects are suspected, but direct measurements are unfeasible.

## Introduction

 Systemic lupus erythematosus (SLE) is a systemic autoimmune disease affecting multiple organs.^1^ The immunopathology is characterised by dysregulated adaptive and innate immune system responses,^2^ resulting in autoantibody production, immune complex formation and complement activation.^3^ Together with genetic predisposition and epigenetic changes, environmental factors underpin the immune dysregulation in SLE.^4^ An example of an environmental factor is synthetic chemicals, which are widespread in the environment, and emerging evidence points towards a role in the development of autoimmunity.^5^

Bisphenol A (BPA) is widely used in the industrial manufacturing of polycarbonate plastics and epoxy resins, for example, in food and water containers, and the migration of BPA monomers into its surroundings contributes to its ubiquitous presence in the environment.^6^ Exposure occurs mainly via oral ingestion, but dermal routes and inhalation are also possible.^7^ Owing to its oestrogen-mimicking effect by binding to oestrogen receptors, BPA is considered a hormone-disrupting xenoestrogen.^6^ However, the molecular mechanisms of action include interference with a wide range of biological receptors, including nuclear and membrane-bound receptors, interaction with transcription factors and induction of epigenetic modifications.^8^

As the role of environmental factors in autoimmune disease is gaining attention, studies suggest that hormone-disrupting chemicals, including BPA, may contribute to the onset and exacerbations of diseases such as SLE.^9^ Exposure to BPA has been associated with immune activation and autoantibody production in SLE mouse models.^10^,^13^ Recently, Wang *et al* described significantly higher levels of BPA in the urine of patients with SLE than in those with controls.^14^ Given that patients with SLE have an activated type I interferon (IFN) system,^15^ increased IFN signalling has been observed in human myeloid cells following BPA exposure.^12^

A mechanism by which environmental factors influence the disease risk is through epigenetic effects, including DNA methylation, histone modifications and non-coding RNAs.^16^ The former is the most studied, and altered DNA methylation status, such as hypomethylated IFN-regulated genes is believed to be important in the pathogenesis of SLE.^17^ BPA exposure has been associated with altered DNA methylation in in vitro, animal and human studies, with a substantial proportion of the human studies being on maternal exposure and foetal effects.^8^ However, so far, BPA-associated DNA methylation changes in SLE have not been investigated.

We hypothesised that BPA exposure can affect SLE pathogenesis via DNA methylation changes. Thus, this study aimed to investigate whether a DNA methylation score based on differential methylation at 5’-C-phosphate-G-3’ (CpG) sites following BPA exposure (BPA-sensitive) is associated with SLE and clinical subphenotypes and compare our results with publicly available gene expression data from BPA-treated cell lines to identify relevant enriched pathways in patients with SLE.

## Methods

### Subjects and samples

The discovery cohort included 347 patients with SLE from Uppsala and Linköping University hospitals and 400 controls from the Uppsala BioResource^18^ among healthy blood donors visiting the Department of Transfusion Medicine, Uppsala University Hospital. The replication cohort consisted of 201 patients with SLE from Karolinska University Hospital and 187 population controls.^17^ Patients fulfilling ≥4 American College of Rheumatology 1982 classification (ACR-82) criteria for SLE were included.^19^ Controls were matched for age and sex. Clinical data were collected from patient charts and included sex, age at sampling, age at diagnosis, disease duration, ACR-82 criteria, autoantibody status, medications at sampling and disease activity at sampling measured as either SLE disease activity index (SLEDAI) or SLE activity measure (SLAM) with high disease activity defined as SLEDAI > 4 or SLAM > 6.^20 21^ Associations between the BPA scores and clinical subphenotypes were analysed in the combined cohort. Clinical information regarding disease manifestations was available for all 548 patients. Medication status included prednisolone, antimalarials, azathioprine, mycophenolate mofetil and methotrexate.

### DNA methylation analysis

Whole blood DNA from patients and controls was subjected to Illumina HumanMethylation 450 k BeadChip methylation array analysis, and in total, methylation levels at 487 577 CpG sites were investigated. Subsequently, signal intensity data were exported to Minfi R package for quality control and subset-quantile within-array normalisation. For methodological details, see online supplemental methods.

### Estimation of the genetic effects of bisphenol A compared with other chemicals

The potential genetic effects of BPA in comparison with other environmental exposures on published SLE genome-wide association study (GWAS) risk genes^22^ were estimated by the Comparative Toxicogenomics Database (CTD) (online supplemental table 1, online supplemental methods).^23^

### Selection of 5’-C-phosphate-G-3’ sites and determination of interferon regulation status

**Figure 1:**
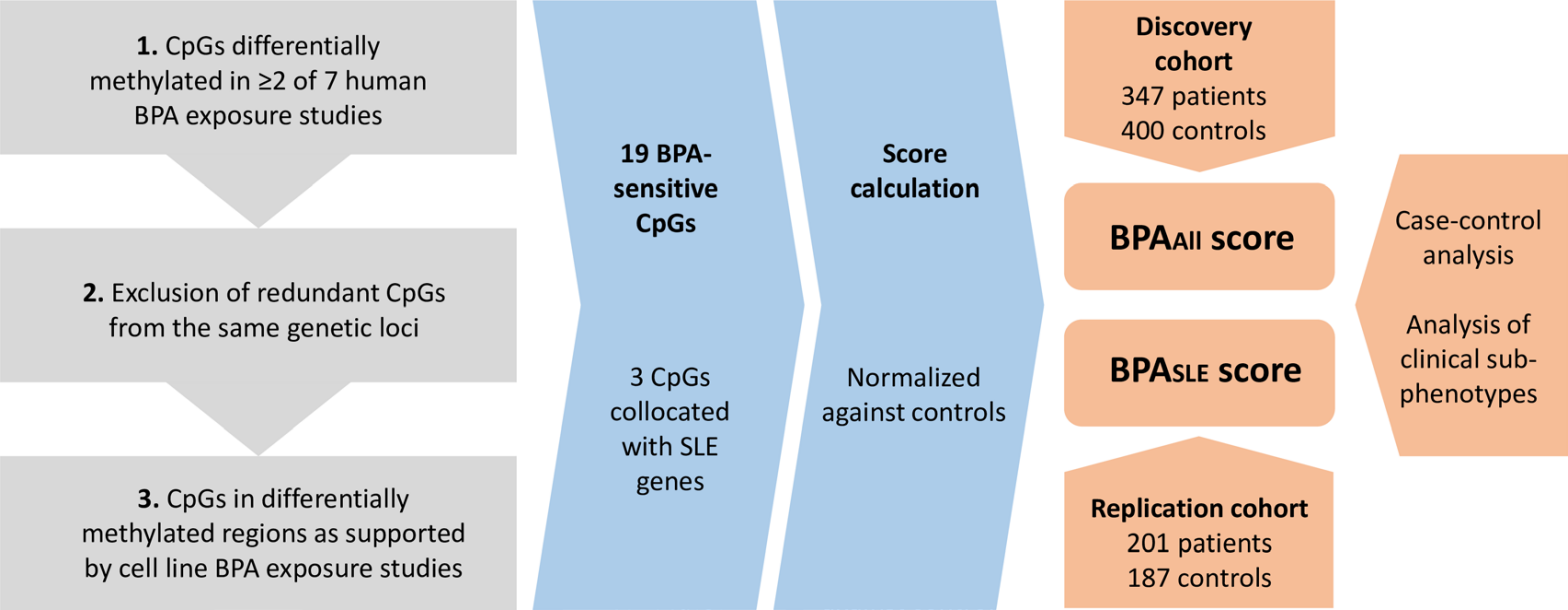
Method overview of the CpG selection strategy, score calculation and cohort analysis. Redundant CpG sites were defined as CpG sites from the same genetic locus. Co-localisation with SLE genes was defined as CpG sites located within 50 kilobase pairs from SLE single nucleotide variants or with GREAT-annotated genes (https://great.stanford.edu/great/public/html/index.php, default settings) overlapping with GWAS catalogue SLE risk genes. To calculate the score, the mean and SD of the methylation β value for each CpG site in the control group were used to achieve standardised values (Z-scores) for each individual. BPA_All_ score, based on all 19 BPA-associated CpG sites; BPA_SLE_ score based on three of the 19 BPA-associated CpG sites identified as SLE-colocalised. BPA, bisphenol A. CpGs, 5’-C-phosphate-G-3’; SLE, systemic lupus erythematosus

The selection of BPA-sensitive CpG sites followed a three-step procedure (figure 1). For details, see online supplemental methods. First, to minimise potential batch effects and study biases, BPA-sensitive CpG sites were identified from seven human BPA exposure studies using the same Illumina methylation array for the analysis of methylation levels in the individuals included in the present study (online supplemental table 2). CpG sites reported as differentially methylated according to authors’ criteria in ≥2 of the selected studies were identified (n=158 CpG sites, online supplemental table 3). Furthermore, 10 redundant CpG sites (ie, from the same genetic locus, located within 50 kilobase (kb) from each other) were excluded, and the first one by coordinates was selected as representative for the locus. Hence, the BPA-sensitive CpG sites included in the scores were identified independently of SLE. Genes were annotated based on the distance to the transcription start sites (TSS) using the GREAT gene annotation service (http://great.stanford.edu/ with default parameters).

For further filtering, effects of BPA treatment/exposure on DNA methylation patterns in cell lines were used to select CpG sites using the Dependency Map (DepMap) portal^24^ including methylation data from 21,337 TSS regions in 419 cancer cell lines. CpG sites with significant DepMap-based TSS methylation changes in any of the annotated genes shown to have quality-controlled filtered methylation data in the SLE cohort were selected. This approach rendered a set of 19 potential BPA-sensitive regions from the Illumina 450 k array. Thereafter, we investigated whether any of the 19 selected BPA-sensitive CpG sites were located adjacent to SLE risk genes by comparing the locations of the BPA-sensitive CpG sites with positions of published GWAS SLE-associated genetic risk loci.^22^ Three CpG sites were determined to be located within 50 kb pairs from SLE single nucleotide variants or with GREAT-annotated genes overlapping with GWAS catalogue SLE genes. These three CpG sites will henceforth be referred to as SLE-co-localised (online supplemental methods). Finally, CTD-reported BPA methylation effects for the genes annotated to the 19 BPA-sensitive CpG sites were investigated (data for all species included). Because IFN plays a role in SLE pathogenesis, we determined whether genes annotated to BPA-sensitive CpG sites were IFN-regulated using data from Interferome, a database including curated IFN regulation data for genes.^25^ For this analysis, all default search conditions were selected, except for species being limited to only human data.

### Calculation of bisphenol A methylation scores

The mean and SD of the methylation β value for each CpG site in the control group were used to achieve standardised values (Z-scores) for each individual (online supplemental methods): Score = (beta value_individual_ – mean_controls_) / standard deviation_controls_). When dissecting the methylation status of the included CpG sites, the majority (13 out of 19 CpG sites) were reported as hypomethylated in the original study or DepMap (online supplemental table 4). To make the scores more interpretable, negative Z-scores from hypomethylated CpG sites were multiplied by −1 if hypomethylated in the same direction as in the original human BPA studies. If the CpG sites from the original studies were significantly affected by BPA, but could be either hypermethylated or hypomethylated, these sites were included as BPA-sensitive and methylation direction for the score construction was selected according to DepMap methylation data. The final methylation scores were obtained through summation of the Z-scores. Two BPA methylation scores were calculated for each individual: BPA_all_ based on all 19 BPA-sensitive CpG sites and BPA_SLE_ based on the three SLE-colocalised BPA-sensitive CpG sites, respectively.

### Comparison of DNA methylation profiles for bisphenol A, estradiol and two common medications

The DepMap portal was assessed to acquire TSS DNA methylation data from cell lines treated with BPA, estradiol, hydroxychloroquine (HCQ) and prednisolone. For each substance, DNA methylation data for 21 337 TSS regions in 419 cell lines were available (online supplemental methods).^24^

### Calculation of transcriptomic changes in bisphenol A-treated samples using publicly available cell line data

Gene expression was analysed using publicly available transcriptomic data from cell lines and human Epstein-Barr virus-transformed B-cells (lymphoblastoid cells). GEO2R (https://www.ncbi.nlm.nih.gov/geo/geo2r/) was used to recalculate differential gene expression between BPA-treated samples and controls (online supplemental methods). Then, MSigDB (https://www.gsea-msigdb.org/gsea/msigdb/index.jsp) and PANTHER (pantherdb.org) were used to identify enriched hallmark signatures and pathways for each cell line (online supplemental methods). Differentially expressed genes in cell lines were compared with genes enriched in blood and immune cells, as reported in the Protein Atlas database (https://www.proteinatlas.org/), and enriched hallmark signatures were identified. Finally, the differentially expressed genes in lymphoblastoid cells were assessed for enriched hallmark signatures and Reactome pathways (https://reactome.org/).

### Statistical analysis

Univariable and multivariable logistic regression models were applied to assess associations between the scores and different binary dependent variables, including SLE disease status and disease manifestations. Differences between different groups in the descriptive data sets were tested with Student’s t-test and Mann–Whitney U test for continuous variables and categorical data with the χ2 test. Principal component analysis and Pearson’s correlation were used to compare the methylation profiles of different substances. Statistical analyses were performed using SPSS version 28.0.1.0^26^ and R studio.^27^ Results with p<0.05 were considered significant.

### Ethical considerations

Ethical approval has been obtained for both patients and controls (DNR 2009/013, 2016/155 and 2020–05065). All study participants gave their informed consent.

## Results

### Bisphenol A is one of the top systemic lupus erythematosus risk gene-interacting compounds in the Comparative Toxicogenomics Database

Initially, gene–chemical interactions of published SLE GWAS risk genes were investigated according to the CTD. For 152 (77%) of the 198 analysed genes, BPA was found among the top 10 gene-interacting chemicals (online supplemental table 1). Interestingly, for 50 (25%) of the SLE risk genes, BPA was the top interacting compound.

### BPA methylation scores associate with systemic lupus erythematosus

Then, a BPA methylation score was calculated for each individual based on the identified 19 BPA-sensitive CpG sites (BPA_All_ score) and a score based on three of these sites that were co-localised with published SLE risk genes (BPA_SLE_ score) (online supplemental table 4, online supplemental methods). The BPA scores were compared between patients and controls. Patients with SLE had significantly higher BPA_All_ score than controls in the discovery cohort (p=2.3×10^-3^) but not in the replication cohort (p=0.12) (table 1online supplemental figure 1). However, the difference between patients with SLE and controls was significant in the meta-analysis (p=7.0×10^-4^). To further explore the potential BPA sensitivity of the selected CpG sites, data on BPA-annotated methylation effects were extracted from CTD. CTD-reported effects of BPA on DNA methylation levels were found for 16 out of 19 genes annotated to BPA-sensitive CpG sites (online supplemental table 4).

**Table 1:** Case–control comparison of bisphenol A scores.

	Discovery cohort		Replication cohort		Meta analysis	
	OR (95% CI)	p	OR (95% CI)	p	OR (95% CI)	p
BPA_All_	1.05 (1.02 to 1.08)	2.3×10^–3^	1.04 (0.99 to 1.09)	0.12	1.05 (1.02 to 1.07)	7.0×10^–4^
BPA_SLE_	1.34 (1.24 to 1.46)	4.6×10^–13^	1.28 (1.15 to 1.43)	1.1×10^–5^	1.32 (1.24 to 1.41)	3.3×10^–17^

Simple logistic regression models, SLE/controls, adjusted for sex and age at sampling. The discovery cohort includes 347 patients and 400 controls. The replication cohort includes 201 patients and 187 controls.

BPA, bisphenol A; SLE, systemic lupus erythematosus.

**Figure 2:**
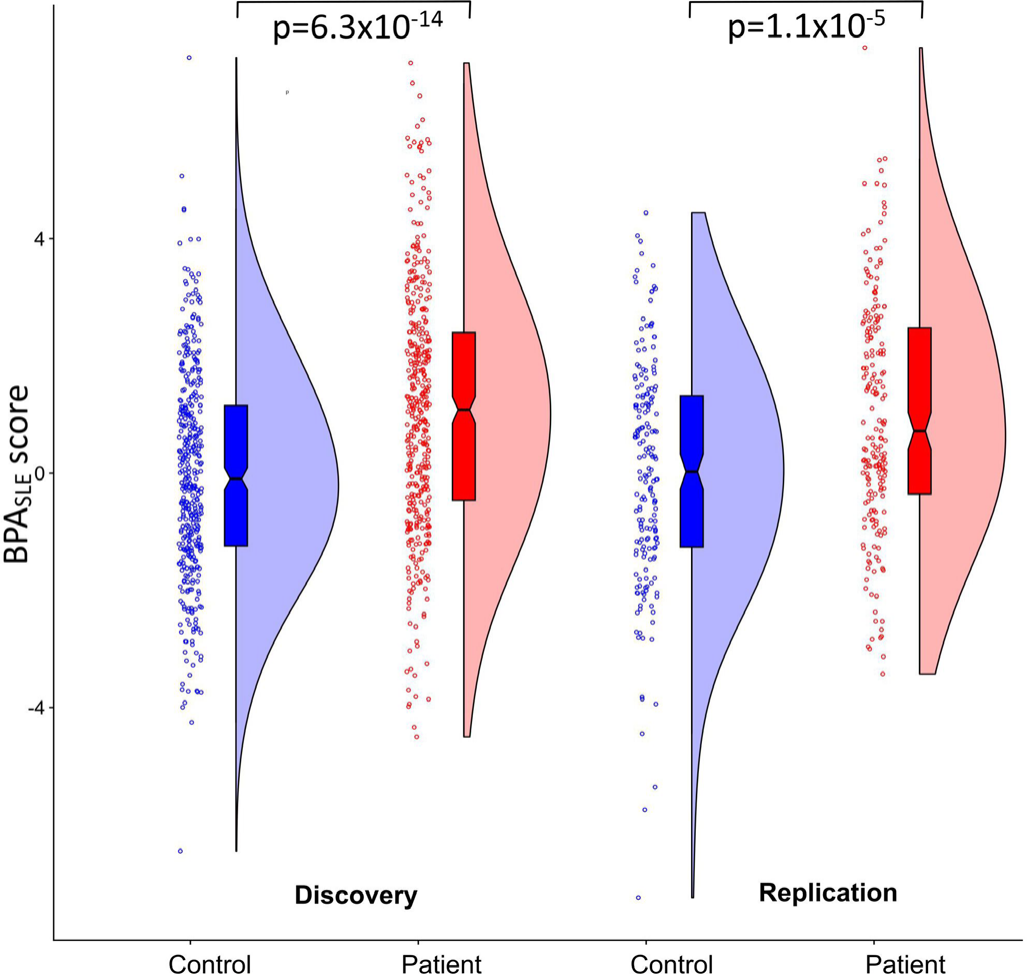
Distribution of the BPA_SLE_ score in patients and controls in the discovery and replication cohorts. Differences in the BPA_SLE_ score between patients and controls were assessed using a two-sided Mann–Whitney U test, with p<0.05 defining significance. BPA, bisphenol A; Discovery, discovery cohort; Replication, replication cohort; SLE, systemic lupus erythematosus.

Case–control analyses were performed for the BPA_SLE_ score. The BPA_SLE_ score was significantly higher in patients with SLE than in controls both in the discovery (p=4.6×10^-13^) and replication (p=1.1×10^-5^) cohorts and meta-analysis (p=3.3×10^-17^) (table 1 and figure 2). All three SLE-co-localised CpG sites were identified as hypomethylated in the original study or DepMap (online supplemental table 4). BPA methylation effects on annotated genes were reported in CTD for two of these CpG sites, including the genes friend leukaemia integration 1 (*FLI1*) and major histocompatibility complex (MHC) class 1 polypeptide-related sequence B (*MICB*) (online supplemental table 4). BPA was the second-highest interacting compound for methylation in CTD when combining the two genes (online supplemental figure 2).

Simple logistic regression models for SLE/controls were adjusted for sex and age at sampling. The discovery cohort included 347 patients and 400 controls. The replication cohort included 201 patients and 187 controls.

### Patient characteristics and associations with the bisphenol A methylation scores

Anti-nuclear antibodies (ANA) were detected in 98% of the patients, and the most common disease manifestation was arthritis, whereas 33% of the patients had a history of SLE nephritis. Detailed clinical data and basic characteristics of the discovery and replication cohorts separately are available in online supplemental tables 5,6. Higher BPA_SLE_ score was associated with serositis, anti-RNP antibodies and presence of three or more autoantibodies (table 2). The BPA_All_ score was not significantly associated with any of the clinical subphenotypes (online supplemental table 7). Both higher BPA_SLE_ and BPA_All_ scores were associated with prednisolone treatment (table 3). Further, a higher BPA_SLE_ score was associated with methotrexate treatment or treatment with any of the disease-modifying anti-rheumatic drugs (DMARDs), whereas a lower BPA_SLE_ score was associated with antimalarial treatment (table 3).

**Table 2:** Associations between the BPA_SLE_ score and clinical subphenotypes

		BPA_SLE_ score	
	N(%)	OR (95% CI)	p
Sex (female)	475 (86.7)	0.95 (0.85 to 1.07)	0.43
Age at sampling ≥60	145 (26.5)	1.00 (0.91 to 1.10)	0.98
ACR criteria*			
Malar rash	291 (53.1)	0.97 (0.89 to 1.05)	0.44
Discoid lupus	110 (20.1)	0.96 (0.87 to 1.06)	0.43
Photosensitivity	337 (61.5)	0.94 (0.87 to 1.02)	0.15
Oral ulcers	115 (21.0)	0.97 (0.88 to 1.08)	0.60
Arthritis	420 (76.6)	1.04 (0.95 to 1.15)	0.39
Serositis	227 (41.4)	**1.10 (1.02 to 1.20)**	**0.019**
Glomerulonephritis	180 (32.8)	1.00 (0.92 to 1.09)	0.98
Neurologic disorder	41 (7.5)	1.05 (0.90 to 1.23)	0.51
Haematological disorder	351 (64.1)	0.97 (0.89 to 1.05)	0.46
Immunological disorder	347 (63.3)	1.03 (0.95 to 1.12)	0.48
Antinuclear antibodies	538 (98.2)	0.99 (0.73 to 1.34)	0.96
Anti-SSA	240 (44.4)	1.00 (0.92 to 1.09)	0.95
Anti-SSB	141 (26.1)	1.01 (0.92 to 1.11)	0.79
Anti-RNP	170 (31.4)	**1.11 (1.01 to 1.21)**	**0.027**
Anti-Sm	74 (13.7)	1.05 (0.93 to 1.18)	0.41
Anti-dsDNA	327 (60.4)	1.01 (0.93 to 1.10)	0.80
Sum ab≥3†	62 (11.5)	**1.10 (1.01 to 1.21)**	**0.037**
SLAM>6 or SLEDAI>4‡	154 (28.4)	1.02 (0.94 to 1.12)	0.61

Simple logistic regression.

Unadjusted p-values are presented with p<0.05 marked in bold.

* Disease manifestations according to the American College of Rheumatology 82-criteria for SLE (ACR-82).

† Sum anti-SSA, anti-SSB, anti-RNP, anti-Sm and anti-dsDNA ≥3.

‡ High disease activity defined as SLE activity measure (SLAM) >6 or SLE disease activity index (SLEDAI) >4.^20 21^

ACR-82, American College of Rheumatology 82-criteria for systemic lupus erythematosus; Anti-dsDNA, anti-double-stranded DNA ; Anti-RNP, anti-ribonucleoprotein antibodies; Anti-Sm, anti-Smith antibodies; Anti-SSA, anti–Sjögren’s-syndrome-related antigen A antibodies; Anti-SSB, anti-Sjögren's syndrome type B antibodies; BPA, bisphenol A; SLAM, systemic lupus erythematosus activity measure; SLE, systemic lupus erythematosus; SLEDAI, systemic lupus erythematosus disease activity index.

**Table 3:** Associations between the BPA scores and medications used

	N(%)	BPA_All_		BPA_SLE_	
		OR (95% CI)	p	OR (95% CI)	p
Pred	326 (59.6)	**1.07 (1.03 to 1.11)**	**3.7×10** ^**–4**^	**1.28 (1.17 to 1.40)**	**9.06×10** ^**–8**^
AM	239 (51.2)	0.98 (0.94 to 1.01)	0.22	**0.91 (0.83 to 1.00)**	**0.041**
AZA	92 (18.4)	0.99 (0.95 to 1.04)	0.81	1.06 (0.95 to 1.19)	0.26
MMF	54 (10.3)	1.04 (0.98 to 1.11)	0.16	**1.01 (0.88 to 1.16)**	**0.86**
MTX	30 (5.8)	1.03 (0.95 to 1.11)	0.47	**1.24 (1.04 to 1.49)**	**0.016**
DMARDs	173 (35.3)	1.02 (0.98 to 1.06)	0.31	1.10 (1.00 to 1.20)	0.041

Simple logistic regression.

Unadjusted p-values, p<0.05 in bold.

AM, antimalarials; AZA, azathioprine; DMARDs, disease-modifying anti rheumatic drugs; MMF, mycophenolate mofetil; MTX, methotrexate; Pred, prednisolone.

### Differences in methylation profiles between two medications, BPA and estradiol

**Figure 3:**
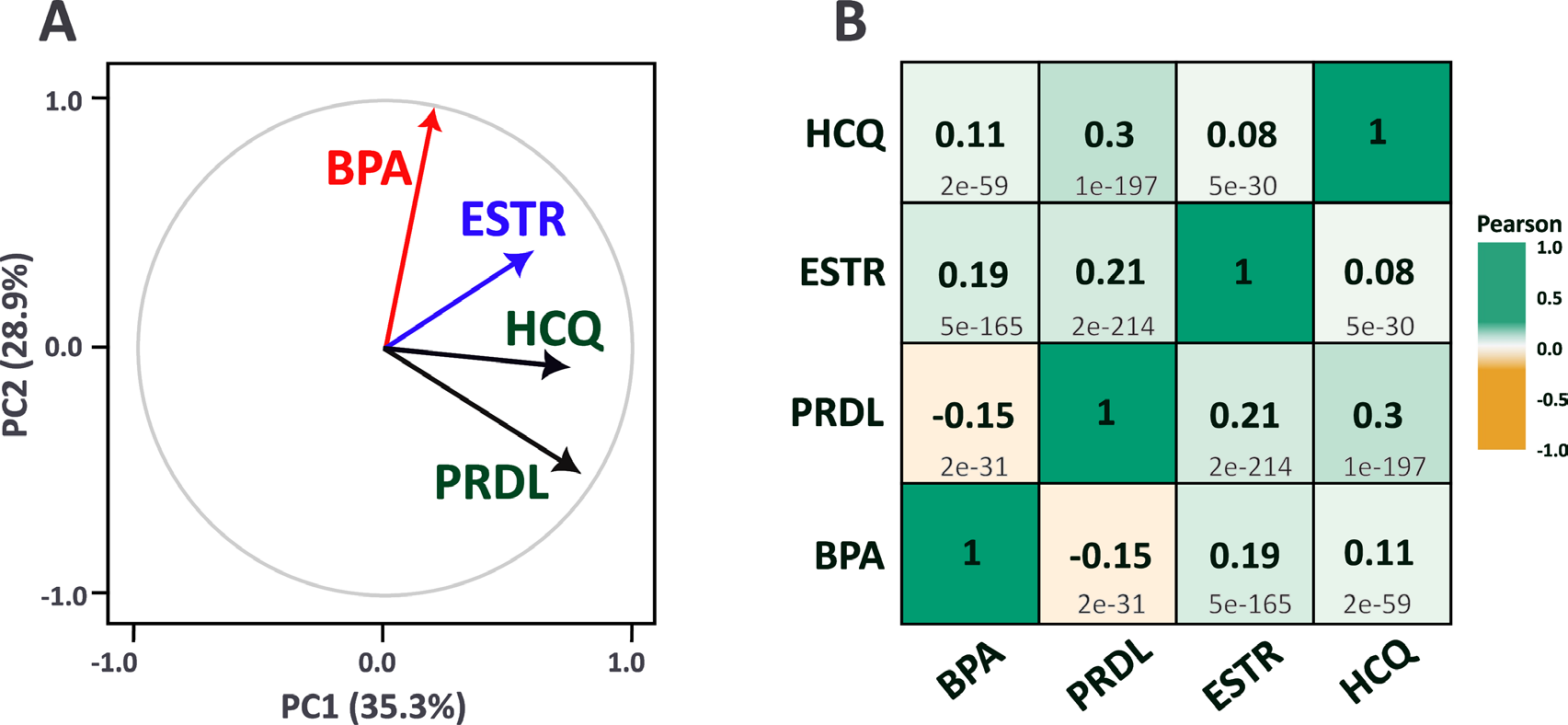
Analysis of DNA methylation profiles for bisphenol A, estradiol, hydroxychloroquine and prednisolone using Dependency Map (DepMap) portal data.^24^ Analysed values include Pearson’s correlation coefficients between exposures/treatments and transcription start site (TSS) methylation for each compound. All DepMap TSS regions (n=21 337) with methylation data based on all available cell lines (n=419) were included. A: PC1 (35.3%) and PC2 (28.9%), first two principal components with percentage of explained variance for each one. The red vector represents BPA; blue vector, estradiol (ESTR); and black vectors, hydroxychloroquine (HCQ) and prednisolone (PRDL). B: Pearson’s correlation matrix of the methylation profiles of the same substances using DepMap data. Pairwise comparisons between compounds are shown. Pearson’s correlation coefficients are indicated in bold and p-values in light grey. BPA, bisphenol A; ESTR, estradiol; HCQ, hydroxychloroquine; PRDL, prednisolone.

Given the association between prednisolone treatment and high BPA scores, we further explored the DNA methylation profile of prednisolone in comparison with the BPA methylation signature.^24^ For each compound, correlation coefficients between exposure/treatment and TSS DNA methylation at 21 337 genes in 419 cell lines were analysed in a Pearson’s correlation matrix and a principal component analysis. In addition to BPA and prednisolone, the endogenous oestrogenic compound estradiol and the frequently prescribed medication HCQ were included. The vector of the first two principal components for estradiol and BPA points in the same direction, whereas the vectors of the prednisolone and HCQ groups together point in another direction (figure 3A). Similarly, the highest positive correlation with BPA was observed for estradiol, and the direction of correlation between BPA and prednisolone was negative. All correlations were significant. HCQ exhibited the highest positive correlation with prednisolone (figure 3B).

### Analysis of publicly available transcriptomic data for bisphenol A-treated cells

**Figure 4:**
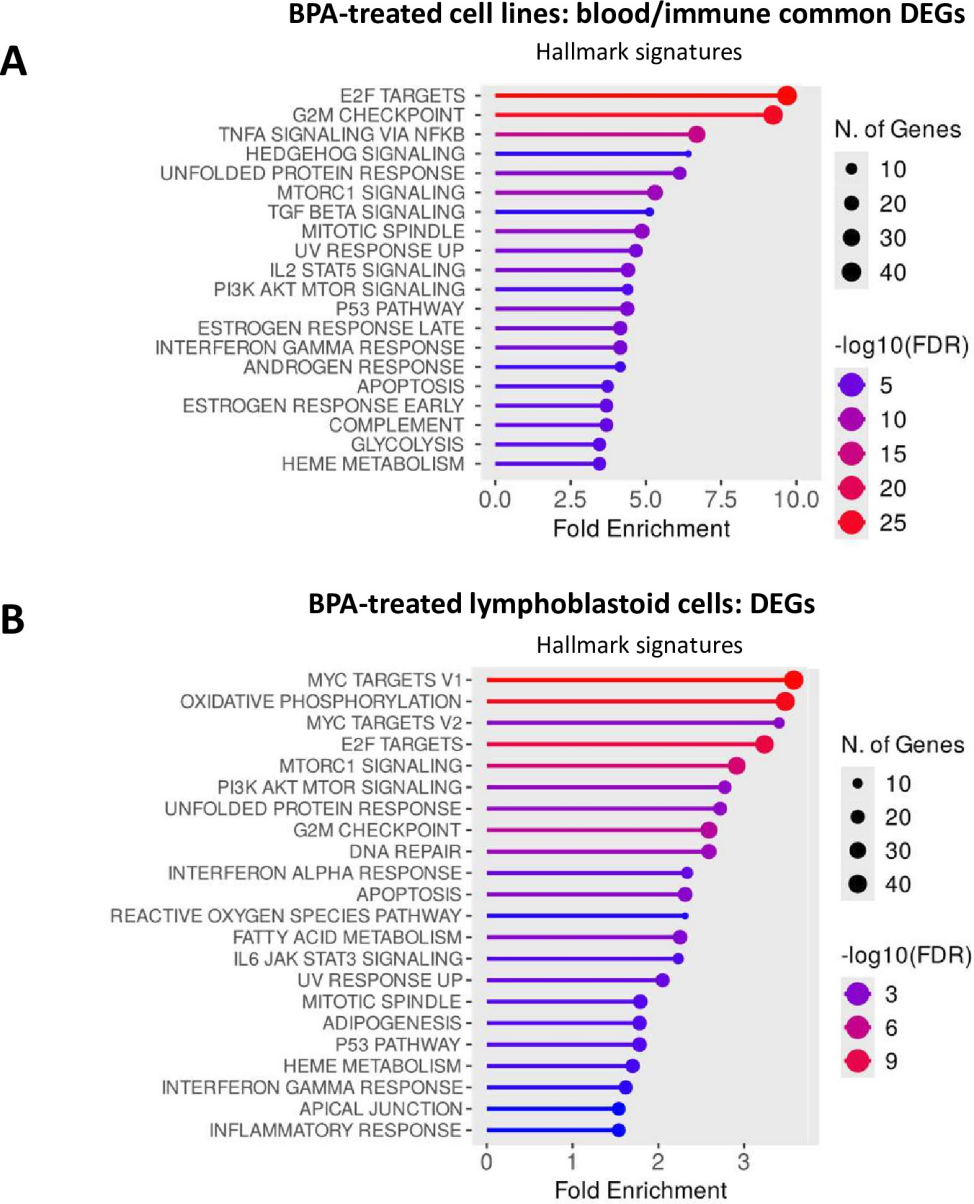
Functional patterns of transcriptomic changes in BPA-treated cells. Hallmark signature enrichment is shown with the length of lines indicating fold enrichment, dot size corresponding to the number of genes and colour indicating the negative logarithm with base 10 of the false discovery rate (-log10(FDR)). A. Functional enrichment for the selected subset of common differentially expressed genes in BPA-treated cell lines that were also enriched in blood and/or immune cells according to https://www.proteinatlas.org/humanproteome/. B. Functional enrichment of differentially expressed genes in BPA-treated lymphoblastoid cells. The hallmark enrichment plots are shown based on the ShinyGO service (https://bioinformatics.sdstate.edu/go/). DEGs, differentially expressed genes; FDR, false discovery rate; IL2, interleukin-2; MTOR, mechanistic target of rapamycin; NFKB, nuclear factor κB; PI3K, phosphatidylinositol 3-kinase; TGF, transforming growth factor

To further explore the effects of BPA on cell signalling, the results of this study on the epigenetic profile of BPA were compared with publicly available transcriptomic data from BPA-treated cells. First, we searched for common affected genes, which were shown to be differentially expressed in at least 2 of 4 diverse cell lines subjected to similar BPA treatments. Several enriched pathways were identified in the analysis, such as IFN-γ signalling, B-cell activation, p38 mitogen-activated protein kinase (MAPK) pathway, transforming growth factor-β (TGF-β) signalling and Notch signalling (online supplemental figure 3). We also investigated enriched gene hallmark signatures according to MSigDB. Enriched signatures found in all cell lines included TGF-β signalling and tumour necrosis factor alpha (TNF-α) signalling via nuclear factor kappa B (NF-κB). Inflammatory response and NOTCH signalling gene signatures were enriched in two of the four cell lines (online supplemental figure 4). To assess the immune relevance of the BPA-affected genes that were differentially expressed in ≥2 of 4 cell lines, we filtered for genes enriched in blood and/or immune cells based on Protein Atlas data. Results showed enrichment of immune-related pathways, notably IFN-γ and inflammation signatures, as well as oestrogen-related responses (figure 4a).

Given the important role of B cells in the pathogenesis of SLE, we analysed BPA-induced transcriptomic changes in human lymphoblastoid cells from healthy donors. This analysis revealed significant enrichment of IFN (alpha and gamma) as well as inflammatory responses (figure 4b). Reactome pathway analysis further highlighted changes in key immunological pathways, including MHC antigen processing, B-cell receptor signalling and interleukin-12 signalling (online supplemental figure 5). With reference to the observed enrichment for IFN signalling, we queried the database Interferome^25^ for detailed information about IFN regulation status and IFN type for the BPA-annotated genes (online supplemental table 4). Out of the 29 genes that were annotated for the 19 BPA-sensitive CpG sites (using GREAT service), 23 (79%) were reported as IFN-regulated. Among them, 14 (48%) were type I IFN-regulated, 20 (69%) were type II IFN-regulated and one (3%) was type III IFN-regulated (online supplemental methods and online supplemental table 8). Annotated genes from all three SLE-co-localised CpG sites were IFN-regulated, with *FLI1* being type II IFN-regulated and *ZMYND15*, *MICB* and *HLA-B* being both type I and type II IFN-regulated.

Finally, we investigated if any of the BPA-annotated genes were also differentially expressed in the four BPA-treated cell lines and lymphoblastoid cells. Among the genes found to both neighbour BPA-sensitive CpG sites and exhibit differential expression, the most significant differentially expressed gene was Mitogen-Activated Protein Kinase Kinase Kinase 9 (*MAP3K9*), which was also differentially expressed in BPA-exposed lymphoblastoid cells. One of the genes annotated to an SLE-co-localised CpG site, *MICB*, was differentially expressed in both the cell lines and lymphoblastoid cells. In total, 10 of the BPA-annotated genes were differentially expressed in the cell lines, and eight were differentially expressed in lymphoblastoid cells (online supplemental table 9).

## Discussion

This study is the first to demonstrate an association between BPA-sensitive DNA methylation scores and SLE, suggesting that BPA-induced epigenetic alterations may contribute to disease pathogenesis. As a xenoestrogen, BPA structurally mimics endogenous oestrogen and may modulate immune function in an oestrogen-like manner, potentially influencing the risk for autoimmune diseases such as SLE.^28^ Consequently, epigenetic changes might comprise one pathophysiological mechanism that bridges the gap between studies reporting associations between BPA and SLE and experimental findings of immune activation and DNA methylation changes following BPA exposure in vitro and in vivo.^8 9^ This mechanism may also be relevant to other autoimmune disorders, as accumulating evidence suggests a role of BPA in conditions such as multiple sclerosis, autoimmune thyroid disease, inflammatory bowel disease, type 1 diabetes and Sjögren’s disease.^9^ Overall, this study highlights the putative role of environmental xenoestrogens in the epigenetic risk of autoimmunity. This insight is particularly relevant for SLE, given the well-established role of oestrogen in its pathogenesis.

Three of the BPA-sensitive CpG sites in this study were localised at known risk genes for SLE; therefore, we investigated these CpG sites in a separate score. Indeed, a stronger association was observed for the BPA_SLE_ score when compared with the BPA_All_ in both the discovery and replication cohorts. Two of the genes annotated to a BPA-sensitive CpG site were *FLI1* and *MICB*. FLI1 is a transcription factor, is an important regulator of several chemokines and cytokines, and has been implicated in nephritis in lupus mouse models.^29^ MICB is a molecule that activates natural killer cells and co-stimulates T cells by binding to the natural killer group 2 receptor.^30^ Altered serum levels of MICB in SLE and in active disease in juvenile SLE have been demonstrated.^31 32^ Altogether, the immunoregulatory and suggested disease-aggravating properties of FLI1 and MICB are in line with data reporting immune activation following BPA exposure in animal SLE models.^10^,^13^

Moreover, BPA score associations with clinical subphenotypes of SLE were investigated. A higher BPA_SLE_ score was associated with serositis. This finding is noteworthy, as aggravated pericarditis has been described in a mouse model following BPA exposure.^33^ Moreover, a higher BPA_SLE_ score was associated with anti-RNP antibodies and presence of multiple autoantibodies. This aligns with previous findings from mouse models, where BPA exposure led to increased autoantibody production, supporting a possible link between BPA-related epigenetic alterations and autoimmune activation.^11 13^ No significant association was found between the BPA scores and disease activity. However, both scores were associated with prednisolone treatment, and the BPA_SLE_ score was associated with DMARDs. Prednisolone and DMARD therapy could be considered as pseudomarkers for more severe disease; however, the associations could also be attributed to medication-induced DNA methylation changes. When the methylation effects were compared in cell lines following exposure to BPA and two common medications for SLE, the results indicated different effects. Furthermore, the highest positive correlation with BPA was observed for estradiol. Taken together, a higher BPA methylation score potentially reflecting methylation changes reminiscent of that of estradiol could predispose to certain clinical manifestations and a disease requiring more intense treatment.

To validate the BPA scores using direct BPA exposure data, we analysed publicly available transcriptomic datasets from BPA-treated cells. Notably, we observed enrichment of genes involved in B-cell activation, IFN-γ (type II IFN) signalling, inflammatory responses and TNF-α signalling via NF-κB pathways that are central to immune regulation and strongly implicated in the pathogenesis of SLE.^34^ Hallmark signatures of similar SLE-relevant pathways and oestrogen responses were observed when filtering for genes expressed in blood/immune cells. This aligns with the established role of BPA as an xenoestrogen capable of activating oestrogen-responsive transcriptional programmes.^6^ Furthermore, transcriptomic changes in BPA-treated lymphoblastoid cells demonstrated sensitivity to BPA in both type I and II IFN responses, further supporting that BPA elicits transcriptional programmes relevant to the pathogenesis of SLE. Overall, the findings suggest that BPA exposure may affect immunological functions relevant to SLE on the transcriptional level, highlighting that BPA-induced epigenetic changes could have functional implications for the pathogenesis of the disease.

Determination of the IFN regulation status by Interferome revealed that 69% of the genes were type II IFN-regulated. In line with the findings of the present study, increased production of IFN-γ was detected in human macrophages following BPA treatment.^35^ A considerable proportion of the BPA-annotated genes were also type I IFN-regulated. Interestingly, high levels of proteins involved in type I IFN signalling have previously been reported following BPA treatment in human myeloid cells.^12^ The high proportion of BPA-annotated genes that were IFN-regulated can be compared with that reported in a previous study, estimating 10% of human genes to be IFN-regulated.^36^ One possible mechanism could be that BPA epigenetically affects partly the same genes as IFN, causing an enhanced effect. Indeed, a feedback loop involving oestrogen receptor alpha and IFN has been suggested as a possible mechanism behind the sexual dimorphism observed in SLE.^37^ Further research is needed to decipher the effects of BPA on epigenetic status in relation to gene expression changes.

**Figure 5:**
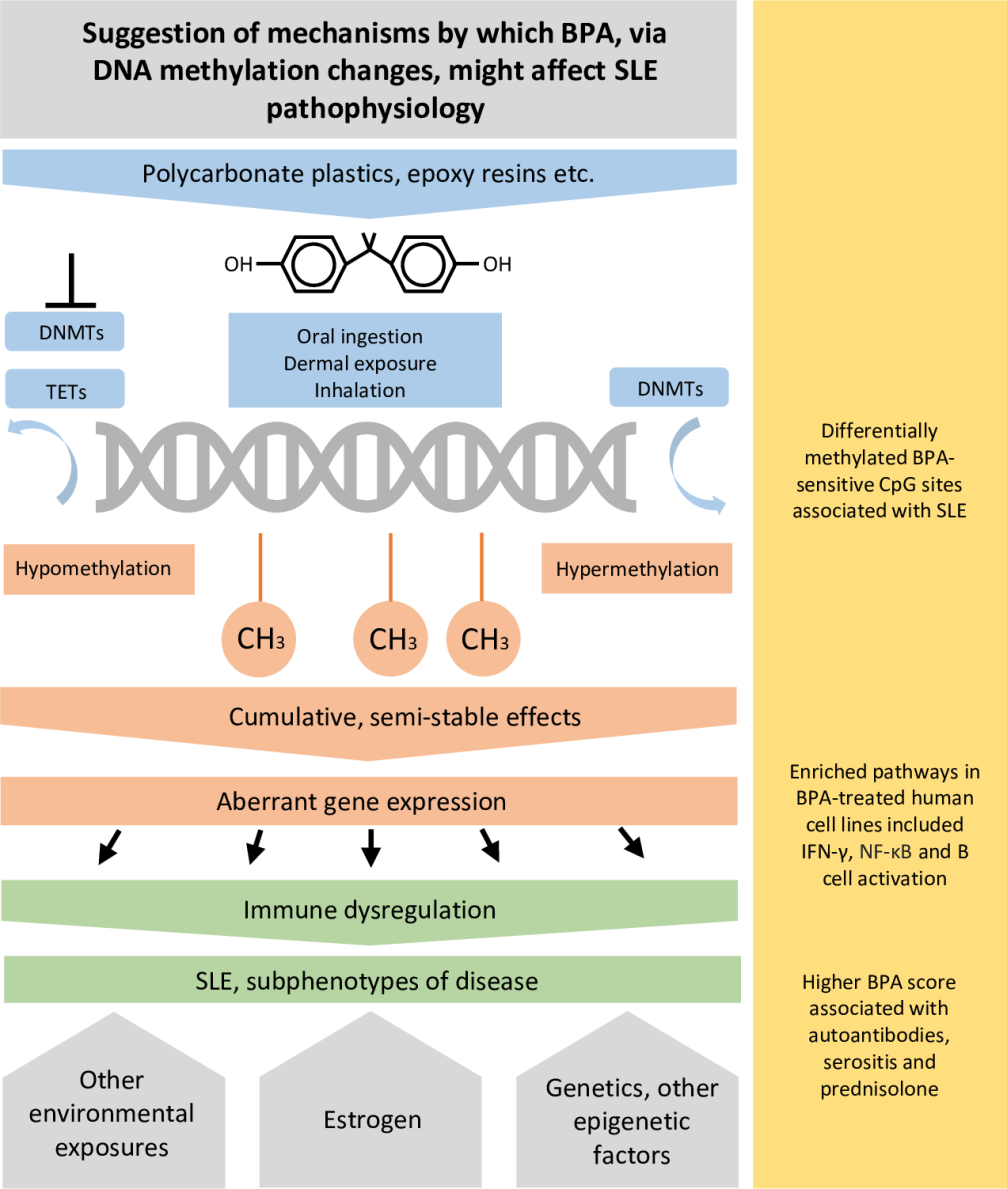
Summary of our suggestion of concept and how the findings of this study might relate to it. BPA-induced DNA methylation changes might mediate aberrant gene expression potentially involving known SLE-relevant immune pathways. Together with other environmental exposures, epigenetic status and genetic factors, these changes might contribute to increased SLE susceptibility and subphenotypes of disease. BPA, bisphenol A; DNMTs, DNA methyltransferases; IFN-ү, interferon gamma; NF-kB, nuclear factor-kB

We found 10 of the BPA-annotated genes to be differentially expressed in the four cell lines exposed to BPA and eight of them to be differentially expressed in lymphoblastoid BPA-exposed cells. The most significant differentially expressed gene in both analyses was *MAP3K9*, which codes for an enzyme involved in the MAPK cascade. Higher levels of the MAP kinases extracellular signal-regulated kinase (ERK) and c-JUN N-terminal kinase early in the disease course have been reported to correlate with organ damage accrual in patients with SLE,^38^ and BPA treatment of murine macrophages induced ERK phosphorylation together with the activation of NF-κB.^39^ We propose that BPA could induce DNA methylation changes, possibly affecting the expression of genes involved in immune signalling pathways relevant to SLE. A summary of our proposed concept is illustrated in figure 5. Although this study reports relatively small exposure effects, the findings may be clinically relevant since the exposome and epigenome are believed to be important in the pathogenesis of SLE, and BPA is a ubiquitously present chemical with suggested early and cumulative effects on DNA methylation. However, further studies are needed to determine a causal relationship between BPA-induced epigenetic changes and SLE.

The strength of this study stems from the large number of individuals investigated, including 548 clinically well-characterised patients with SLE. Furthermore, the BPA-sensitive CpG sites were identified through a three-step procedure where CpG sites differentially methylated in human BPA exposure studies were selected, and redundant CpG sites were excluded by filtering based on cell line data. Most CpG sites were selected based on fetal exposure; however, other CpG sites might be affected by BPA later in life. However, the selected set of BPA exposure studies is also a strength because in utero exposure is believed to play a role in the future risk of disease via epigenetic effects.^40^ A limitation of the study is the lack of direct BPA measurements. Moreover, the models were not adjusted for cell type proportions, which could be considered a limitation. Nevertheless, cell composition changes due to BPA-induced alterations in methylation pattern itself could be a biological response to exposure.

## Conclusions

We report that BPA methylation scores are associated with SLE and specific clinical subphenotypes, suggesting a potential epigenetic link between BPA exposure and disease expression. Supporting these findings, analysis of BPA-treated cells revealed altered expression of immune-related genes, including genes annotated to CpG sites within the methylation scores. Thus, BPA-induced epigenetic changes may contribute to SLE susceptibility via the aberrant expression of immunoregulatory genes. The approach employed in this study may be broadly applicable to other environmental exposures and disease contexts. Although our results underscore a possible need for precautionary measures to reduce BPA exposure, further studies are needed to clarify the exact role of BPA in SLE pathogenesis.

## Supplementary material

10.1136/rmdopen-2025-006021 online supplemental file 1

10.1136/rmdopen-2025-006021 online supplemental table 1

## Data Availability

Data are available upon reasonable request.
