# Supplementary material for: Patients with systemic lupus erythematosus (SLE) have an increased bisphenol A methylation score linked to SLE risk genes and selected clinical subphenotypes
Source: RMD Open. 2025 Sep 25;11(3):e006021. doi: 10.1136/rmdopen-2025-006021 (PMC12481292; doi:10.1136/rmdopen-2025-006021)
Supplement: online supplemental file 1 [file rmdopen-11-3-s001.pdf]

Supplementary methods

DNA methylation analysis: Analysis of DNA methylation levels at 485,577 CpG sites was performed using the HM450k BeadChip (Illumina Inc., San Diego, CA, USA), a microarray measuring DNA methylation levels at individual CpG sites on bisulfite-converted DNA and thereby generating methylation data of various regions across the genome [1]. Using the EZ DNA Methylation-Gold Kit (Zymo Research, Irvine, CA, USA), 500 ng of DNA was subjected to bisulfite treatment. To avoid batch effects, samples were randomized on the BeadChips. Next, the BeadChips were scanned on an iScan SQ instrument (Illumina), followed by exportation of signal intensities as intensity data files. Quality control (QC) and Subset-quantile Within Array Normalization (SWAN) were performed using the Minfi R package [2-4]. The fraction of the signal intensity from the methylated CpG sites over the total intensity (range 0-1, corresponding to 0-100% methylation) was calculated to achieve methylation β-values.

Estimation of BPA genetic effects compared to other environmental exposures: First, SLE genetic loci were extracted from the current (accessed 11 March 2025) version of the GWAS Catalogue (https://www.ebi.ac.uk/gwas/) [5]. Selection of SLE-associated SNVs was based on all published SLE-associations with a p-value $\leq 5 \times 10^{-8}$. For each associated SNV, the mapped neighboring gene(s) were extracted. Genes reported from at least two studies, i.e. found to be reproducible were selected rendering a set of 198 SLE genes, supplementary table S1. Next, Comparative Toxicogenomics Database (CTD) (https://ctdbase.org/) was used to identify which chemicals have been reported to interact with the SLE genes [6]. Top 10 interacting chemicals with the largest number of annotated gene-chemical interactions were identified for each gene. In the analysis, data for all species were shown and all types of interactions were included. Finally, it was noted for each particular gene whether BPA was among top 10 interacting chemicals and the number of annotated BPA interactions for each gene was listed.

Selection of BPA-sensitive CpG sites: Human BPA exposure DNA methylation data were selected from environmental exposure studies (whole blood, peripheral blood mononuclear cells (PBMCs) or placenta, Illumina platform) and direct BPA treatment of cells were used, rendering a set of seven selected BPA exposure studies [7-13]. Illumina 450k CpG sites reported as differentially methylated based on authors criteria in at least two of the seven studies were selected resulting in a list of 158 CpG sites, supplementary file 2 table S3. For further filtering, redundant CpG sites (i.e. from the same genetic locus, located within 50 kb from each other) were excluded and the first one by coordinates was selected as representative for the locus. Ten redundant CpG sites were excluded. Associated genes were annotated based on the distance to transcription start sites (TSS) using GREAT gene annotation service (https://great.stanford.edu/great/public/html/index.php, default settings) [14]. To further increase potential BPA-sensitivity, effects of BPA treatment/exposure on DNA methylation patterns in cell lines were used to select CpG sites using the Dependency Map (DepMap) portal [15]. CpG sites with significant DepMap-based TSS methylation changes in any of the annotated genes shown to have quality controlled filtered methylation data in the SLE cohort were selected. This approach rendered a set of 19 potential BPA-sensitive regions from the Illumina 450k panel, supplementary file 1 table

S1. The locations of the 19 BPA sensitive CpGs were then compared with positions of published SLE-associated genetic loci according to a genome wide association studies (GWAS) catalog (https://www.ebi.ac.uk/gwas/) [16]. CpG sites located within 50 kilobase pairs from SLE single nucleotide variants (SNVs) or with GREAT-annotated genes overlapping with GWAS catalog SLE genes were selected as SLE-colocalized CpG sites. Three CpG sites were determined to be SLE-colocalized.

Top GWAS hits for selection of SLE-colocalized CpG sites:

**CpG**	**SNV-risk allele**	**GWAS p-value**	**GWAS catalogue study ID**
cg00344445	rs684150-T	$4x10^{-10}$	GCST011956
cg17900689	rs2286672-T	$3x10^{-9}$	GCST003155
cg18204091	rs2263318-A	$1x10^{-19}$	GCST011956

Calculation of BPA methylation scores: The scores were calculated according to the formula developed by Feng X *et al*. [17] and described for DNA methylation data by Björk A *et al.* [18]. The mean and standard deviation (SD) of the methylation β value for each CpG site in the control group were used to achieve standardized values (Z-scores) for each individual:

$Z\text{-score}_{individual}=(\beta\ value_{individual}-mean_{control})/SD_{control}$.

BPA scores were calculated separately in the discovery and replication cohort, i.e. for each individual the score calculations were based on the cohort-specific means. Negative Z score values, i.e. Z score values from hypomethylated BPA-sensitive CpG sites, were multiplied by -1 if differentially methylated in the same direction as in the seven human BPA studies. If CpG sites were BPA-sensitive in original studies with varying hypo- or hypermethylation status, the methylation direction was defined according to DepMap BPA data for associated genes. The final methylation scores were obtained through summation of the Zscores. Two BPA methylation scores were calculated for each individual: BPA_{all} based on all 19 BPA-sensitive CpGs and BPA_{SLE} based on the three SLE-colocalized BPA sensitive CpGs.

Comparison of the methylation profiles of BPA, estradiol and two common medications: Pearson correlation values for each compound between treatment and methylation 1 kb upstream TSS were extracted from the Dependency Map Portal using the Custom analyses tool [15]. Analyzed values included 21337 TSS regions from 419 cell lines.

Selection and analysis of publicly available BPA treatment transcriptomic data:

First, gene expression changes in BPA-treated human cell lines were compared with DMSO-treated control samples using publicly available gene expression profile data. GEO2R (https://www.ncbi.nlm.nih.gov/geo/geo2r/) was used to recalculate differential gene expression between BPA-treated samples and controls from four studies performing transcriptomic profiling of diverse chemical-treated cell lines; Ishikawa, HepG2, Y79

retinoblastoma and MCF 7 [19-21]. In the cell line selection process, cancer cell line datasets available for GEO2R with only Dimethyl sulfoxide (DMSO)-treated controls (i.e. not "untreated" or "water" as a control) were selected and one cell line per cancer type was included, i.e. extra experiments with the same or derived cell lines were excluded. For each dataset, BPA experiment samples were selected regardless of treatment dose.

For the non-cancer immune cells treated with BPA, the experiment investigating lymphoblastoid cells was included. The original B-cells from healthy participants from the 1000 Genomes project were EBV-transformed and further studied for transcriptomic effects of selected chemicals in the GSE207049 study [22]. Since the original study lacked DMSO-treated controls, we recalculated differential gene expression of BPA-treated lymphoblastoid cells in comparison with ethanol-treated cells using GEO2R.

For cancer cell line and lymphoblastoid cells, BPA treatment dose and duration of BPA exposure for the different cells are shown in the table below:

**Study**	**Cell source**	**BPA treatment doses**	**Treatment duration**
Cancer cell lines			
[19]	GSE69849 Ishikawa	1, 10, 100 µM	6, 24 or 48 h
[19]	GSE69850 HepG2	1, 10, 100 µM	6, 24 or 48 h
[20]	GSE146255 Y79 retinoblastoma	40 µM	48 h
[21]	GSE271332_MCF7	0.5, 1, 5, 10, 25 µM	48 h
Lymphoblastoid (EBV-transformed B-cells)			
[22]	GSE207049_lymphoblastoid	6 ng/µl, total 30 ng per sample	4 h

Only experiments with "Bisphenol A" without any modifications were included. Calculations of differential gene expression between BPA-treated and control samples were performed with default settings (Benjamini & Hochberg False discovery rate, significance level cut off 0.05, Log2 fold change threshold 0) and with all doses grouped together.

Selection of blood and immune-relevant genes from cell line transcriptomic data

In the cancer cell line BPA-treated transcriptomic data we selected differentially expressed genes (DEGs), annotated as being significantly differentially expressed in at least two from the four selected cell line experiments.

To enhance the relevance of our findings to the autoimmune disease SLE, we refined the list of DEGs by selecting genes with documented elevated expression in human blood and

immune cells. This selection was based on published dataset of human genes, characterized with an elevated expression in any blood & immune cells compared to other cell type groups, according to Protein Atlas study (dataset “The blood & immune cell-specific proteome”, 3234 genes, downloaded from: https://www.proteinatlas.org/humanproteome/single+cell/single+cell+type/Blood+%26+immune+cells) [23].

Functional enrichment analysis

Functional enrichment of the DEGs was estimated using PANTHER pathways for protein families [24, 25],Reactome human-centric pathways for both protein-coding and RNA genes [26] human Hallmark gene sets from Molecular Signatures Database (MSigDB) [27-29].

For graphical demonstration of the pathway enrichment, ShinyGO (default parameters, including 0.05 FDR cutoff and allowed pathway size ranging from 2 to 5000) was used (https://bioinformatics.sdstate.edu/go/) [30].

References

1. Bibikova M, Barnes B, Tsan C, Ho V, Klotzle B, Le JM, et al. High density DNA methylation array with single CpG site resolution. Genomics. 2011;98(4):288-95.
2. IBM Corp. Released 2021. IBM SPSS Statistics for Windows, Version 28.0.: Armonk, NY: IBM Corp.
3. Aryee MJ, Jaffe AE, Corrada-Bravo H, Ladd-Acosta C, Feinberg AP, Hansen KD, et al. Minfi: a flexible and comprehensive Bioconductor package for the analysis of Infinium DNA methylation microarrays. Bioinformatics. 2014;30(10):1363-9.
4. Maksimovic J, Gordon L, Oshlack A. SWAN: Subset-quantile within array normalization for illumina infinium HumanMethylation450 BeadChips. Genome Biol. 2012;13(6):R44.
5. Cerezo M, Sollis E, Ji Y, Lewis E, Abid A, Bircan KO, et al. The NHGRI-EBI GWAS Catalog: standards for reusability, sustainability and diversity. Nucleic Acids Res. 2025;53(D1):D998-d1005.
6. Davis AP, Wiegers TC, Sciaky D, Barkalow F, Strong M, Wyatt B, et al. Comparative Toxicogenomics Database's 20th anniversary: update 2025. Nucleic Acids Res. 2025;53(D1):D1328-d34.
7. Miura R, Araki A, Minatoya M, Miyake K, Chen M-L, Kobayashi S, et al. An epigenome-wide analysis of cord blood DNA methylation reveals sex-specific effect of exposure to bisphenol A. Scientific Reports. 2019;9(1):12369.
8. Song X, Wang Z, Zhang Z, Miao M, Liu J, Luan M, et al. Differential methylation of genes in the human placenta associated with bisphenol A exposure. Environ Res. 2021;200:111389.
9. Huang YF, Chang CH, Chen PJ, Lin IH, Tsai YA, Chen CF, et al. Prenatal Bisphenol a Exposure, DNA Methylation, and Low Birth Weight: A Pilot Study in Taiwan. Int J Environ Res Public Health. 2021;18(11).
10. Awada Z, Nasr R, Akika R, Cahais V, Cuenin C, Zhivagui M, et al. DNA methylome-wide alterations associated with estrogen receptor-dependent effects of bisphenols in breast cancer. Clin Epigenetics. 2019;11(1):138.
11. Otsuka S, Qin XY, Wang W, Ito T, Nansai H, Abe K, et al. iGEM as a human iPS cell-based global epigenetic modulation detection assay provides throughput characterization of chemicals affecting DNA methylation. Sci Rep. 2023;13(1):6663.

12. Sol CM, Gaylord A, Santos S, Jaddoe VWV, Felix JF, Trasande L. Fetal exposure to phthalates and bisphenols and DNA methylation at birth: the Generation R Study. Clin Epigenetics. 2022;14(1):125.
13. McCabe CF, Padmanabhan V, Dolinoy DC, Domino SE, Jones TR, Bakulski KM, et al. Maternal environmental exposure to bisphenols and epigenome-wide DNA methylation in infant cord blood. Environ Epigenet. 2020;6(1):dvaa021.
14. McLean CY, Bristor D, Hiller M, Clarke SL, Schaar BT, Lowe CB, et al. GREAT improves functional interpretation of cis-regulatory regions. Nat Biotechnol. 2010;28(5):495-501.
15. Tsherniak A, Vazquez F, Montgomery PG, Weir BA, Kryukov G, Cowley GS, et al. Defining a Cancer Dependency Map. Cell. 2017;170(3):564-76.e16.
16. Cerezo M, Sollis E, Ji Y, Lewis E, Abid A, Bircan Karatuğ O, et al. The NHGRI-EBI GWAS Catalog: standards for reusability, sustainability and diversity. Nucleic Acids Research. 2024;53(D1):D998-D1005.
17. Feng X, Wu H, Grossman JM, Hanvivadhanakul P, FitzGerald JD, Park GS, et al. Association of increased interferon-inducible gene expression with disease activity and lupus nephritis in patients with systemic lupus erythematosus. Arthritis Rheum. 2006;54(9):2951-62.
18. Bjork A, Richardsdotter Andersson E, Imgenberg-Kreuz J, Thorlacius GE, Mofors J, Syvanen AC, et al. Protein and DNA methylation-based scores as surrogate markers for interferon system activation in patients with primary Sjogren's syndrome. RMD Open. 2020;6(1).
19. De Abrew KN, Kainkaryam RM, Shan YK, Overmann GJ, Settivari RS, Wang X, et al. Grouping 34 Chemicals Based on Mode of Action Using Connectivity Mapping. Toxicol Sci. 2016;151(2):447-61.
20. Kim C-H, Kim MJ, Park J, Kim J, Kim J-Y, An M-J, et al. Bisphenol A Exposure Changes the Transcriptomic and Proteomic Dynamics of Human Retinoblastoma Y79 Cells. Genes. 2021;12(2):264.
21. Beal MA, Coughlan MC, Nunnikhoven A, Gagné M, Barton-Maclaren TS, Bradford LM, et al. High-throughput transcriptomics toxicity assessment of eleven data-poor bisphenol A alternatives. Environmental Pollution. 2024;361:124827.

22. Lea AJ, Peng J, Ayroles JF. Diverse environmental perturbations reveal the evolution and context-dependency of genetic effects on gene expression levels. Genome Res. 2022; 32:1826-1839.
23. Shi M, Mear L, Karlsson M, Bueno Alvez M, Digre A, Schutten R et al. A resource for whole-body gene expression map of human tissues based on integration of single cell and bulk transcriptomics. Genome Biol. 2025; 26:152.

24. Thomas PD, Ebert D, Muruganujan A, Mushayahama T, Albou LP, Mi H. PANTHER: Making genome-scale phylogenetics accessible to all. Protein Sci. 2022;31(1):8-22.
25. Mi H, Thomas P. PANTHER pathway: an ontology-based pathway database coupled with data analysis tools. Methods Mol Biol. 2009;563:123-40.

26. Croft D, O'Kelly G, Wu G, Haw R, Gillespie M, Matthews L et al. Reactome: a database of reactions, pathways and biological processes. Nucleic Acids Res. 2011; 39: D691-697.
27. Subramanian A, Tamayo P, Mootha VK, Mukherjee S, Ebert BL, Gillette MA, et al. Gene set enrichment analysis: A knowledge-based approach for interpreting genome-wide expression profiles. Proceedings of the National Academy of Sciences. 2005;102(43):15545-50.
28. Liberzon A, Subramanian A, Pinchback R, Thorvaldsdóttir H, Tamayo P, Mesirov JP. Molecular signatures database (MSigDB) 3.0. Bioinformatics. 2011;27(12):1739-40.
29. Liberzon A, Birger C, Thorvaldsdóttir H, Ghandi M, Mesirov JP, Tamayo P. The Molecular Signatures Database (MSigDB) hallmark gene set collection. Cell Syst. 2015;1(6):417-25.

30. Ge SX, Jung D, Yao R. ShinyGO: a graphical gene-set enrichment tool for animals and plants. Bioinformatics. 2019;36(8):2628-9.
