# Supplementary material for: Patients with systemic lupus erythematosus (SLE) have an increased bisphenol A methylation score linked to SLE risk genes and selected clinical subphenotypes
Source: RMD Open. 2025 Sep 25;11(3):e006021. doi: 10.1136/rmdopen-2025-006021 (PMC12481292; doi:10.1136/rmdopen-2025-006021)
Supplement: online supplemental table 1 [file rmdopen-11-3-s002.pdf]

Supplementary tables and figures

Supplementary table S1. *Top 10 gene-interacting chemicals for each of the 198 identified SLE-associated genes according to Comparative Toxicogenomics Database (https://ctdbase.org/)* [1]. *For methodological details, see supplementary methods.*

No	Mapped SLE GWAS gene	CTD annotation	Bisphenol A: presence among top10 interacting chemicals	Number of bisphenol A interactions	Gene type
1	*ACAP1*	yes	Yes, no.1	6	Protein-coding
2	*AFF1*	Yes	Yes, no.1	9	Protein-coding
3	*ANKRD55*	Yes	Yes, no.1	6	Protein-coding
4	*ANKS1A*	Yes	Yes, no.4	2	Protein-coding
5	*ANXA6*	Yes	Yes, no.1	15	Protein-coding
6	*AP4B1-AS1*	Yes	No	0	Antisense
7	*AP5B1*	Yes	Yes, no.1	3	Protein-coding
8	*ARHGAP31*	Yes	Yes, no.1	6	Protein-coding
9	*ARID5B*	Yes	Yes, no.1	10	Protein-coding
10	*ATG5*	Yes	No	10	Protein-coding
11	*ATP6V1G3*	Yes	Yes, no.3	2	Protein-coding
12	*ATXN2*	Yes	Yes, no.1	6	Protein-coding
13	*BACH2*	Yes	Yes, no.6	3	Protein-coding
14	*BANK1*	Yes	Yes, no.2	4	Protein-coding
15	*BLK*	Yes	Yes, no.2	3	Protein-coding
16	*BLTP3A*	Yes	Yes, no.6	2	Protein-coding
17	*C2*	Yes	Yes, no.6	4	Protein-coding
18	*C4B*	Yes	Yes, no.1	10	Protein-coding
19	*CARMIL1*	Yes	Yes, no.5	3	Protein-coding
20	*CCDC116*	Yes	Yes, no.2	2	Protein-coding
21	*CCHCR1*	Yes	Yes, no.1	8	Protein-coding
22	*CCL22*	Yes	No	3	Protein-coding
23	*CCR3*	Yes	No	1	Protein-coding
24	*CD226*	Yes	Yes, no.1	6	Protein-coding

25	*CD44-DT*	No	No	0	Divergent transcript
26	*CD58*	Yes	No	0	
27	*CDC37*	Yes	Yes, no.2	7	Protein-coding
28	*CDKN1B*	Yes	Yes, no.9	18	Protein-coding
29	*CLEC16A*	Yes	Yes, no.2	7	Protein-coding
30	*CMAHP*	Yes	No	0	Pseudogene
31	*CRHR1*	Yes	Yes, no.9	4	Protein-coding
32	*CTLA4*	Yes	No	1	Protein-coding
33	*CYCSP20*	No	No	0	Pseudogene
34	*CYP21A2*	Yes	No	1	Protein-coding
35	*DDX6*	Yes	Yes, no.1	7	Protein-coding
36	*DGKQ*	Yes	Yes, no.2	4	Protein-coding
37	*DGUOK-AS1*	Yes	No	0	Antisense
38	*DRAM1*	Yes	Yes, no.1	10	Protein-coding
39	*DRD4*	Yes	No	2	Protein-coding
40	*DUSP22*	Yes	Yes, no.3	4	Protein-coding
41	*EIF4H*	Yes	Yes, no.3	5	Protein-coding
42	*ELF1*	Yes	Yes, no.4	4	Protein-coding
43	*ETS1*	Yes	Yes, no.4	7	Protein-coding
44	*FAM167A*	Yes	Yes, no.1	4	Protein-coding
45	*FAM86B3P*	Yes	Yes, no.7	1	Pseudogene
46	*FAP*	Yes	Yes, no.1	6	Protein-coding
47	*FCGR2A*	Yes	Yes, no.1	6	Protein-coding
48	*FCHSD2*	Yes	Yes, no.3	6	Protein-coding
49	*FCRL5*	Yes	Yes, no.10	1	Protein-coding
50	*FLI1*	Yes	Yes, no.2	8	Protein-coding
51	*FRMD4A*	Yes	Yes, no.1	9	Protein-coding
52	*FUT2*	Yes	Yes, no.2	5	Protein-coding
53	*GLS*	Yes	Yes, no.2	10	Protein-coding
54	*GPR19*	yes	Yes, no.3	3	Protein-coding
55	*GRB2*	Yes	Yes, no.2	6	Protein-coding

56	*GTF2I*	Yes	Yes, no.4	6	Protein-coding
57	*GTF2I-AS1*	Yes	No	0	Antisense
58	*GTF2IRD1*	Yes	Yes, no.3	6	Protein-coding
59	*H2BP5*	No	No	0	Pseudogene
60	*H3P5*	No	No	0	Pseudogene
61	*HCP5*	Yes	No	1	Protein-coding
62	*HIP1*	Yes	Yes, no.1	10	Protein-coding
63	*HLA-DPA1*	Yes	Yes, no.2	3	Protein-coding
64	*HLA-DPB1*	Yes	No	0	Protein-coding
65	*HLA-DQA1*	Yes	No	1	Protein-coding
66	*HLA-DQB1*	Yes	No	1	Protein-coding
67	*HLA-DQB1-AS1*	Yes	No	0	Antisense
68	*HLA-DQB2*	Yes	Yes, no.6	1	Protein-coding
69	*HLA-DQB3*	No	No	0	Protein-coding
70	*HLA-DRA*	Yes	No	1	Protein-coding
71	*HLA-DRB1*	Yes	Yes, no.2	3	Protein-coding
72	*HLA-DRB5*	Yes	No	0	Protein-coding
73	*HLA-DRB9*	No	No	0	Protein-coding
74	*ICAM3*	Yes	Yes, no.5	2	Protein-coding
75	*IFIH1*	Yes	Yes, no.4	5	Protein-coding
76	*IKBKE*	Yes	Yes, no.3	6	Protein-coding
77	*IKZF1*	Yes	Yes, no.2	6	Protein-coding
78	*IKZF2*	Yes	Yes, no.1	10	Protein-coding
79	*IKZF3*	Yes	Yes, no.2	5	Protein-coding
80	*IL10*	Yes	Yes, no.5	50	Protein-coding
81	*IL12A-AS1*	Yes	No	0	Antisense
82	*IL12B*	Yes	No	5	Protein-coding
83	*IL12RB2*	Yes	Yes, no.2	3	Protein-coding
84	*IRAK1*	Yes	Yes, no.2	9	Protein-coding
85	*IRF1*	Yes	Yes, no.9	5	Protein-coding
86	*IRF5*	Yes	Yes, no.1	9	Protein-coding
87	*IRF7*	Yes	Yes, no.4	10	Protein-coding

88	*IRF8*	Yes	Yes, no.7	4	Protein-coding
89	*ITGAM*	Yes	No	3	Protein-coding
90	*ITGAX*	Yes	Yes, no.2	9	Protein-coding
91	*IUR1*	No	No	0	Protein-coding
92	*JAK2*	Yes	Yes, no.3	15	Protein-coding
93	*JAZF1*	Yes	Yes, no.4	6	Protein-coding
94	*KCP*	Yes	Yes, no.2	5	Protein-coding
95	*KEAP1*	Yes	No	10	Protein-coding
96	*LBH*	Yes	Yes, no.2	7	Protein-coding
97	*LINC00824*	Yes	No	0	lncRNA
98	*LINC01082*	No	No	0	lncRNA
99	*LINC01149*	Yes	No	0	lncRNA
100	*LINC01845*	Yes	No	0	lncRNA
101	*LINC01967*	No	No	0	lncRNA
102	*LINC02098*	Yes	No	0	lncRNA
103	*LINC02132*	Yes	No	0	lncRNA
104	*LINC02539*	No	No	0	lncRNA
105	*LINC02694*	Yes	No	0	lncRNA
106	*LINC02865*	Yes	No	0	lncRNA
107	*LINC02929*	No	No	0	lncRNA
108	*LINC03004*	No	No	0	lncRNA
109	*LINC03066*	No	No	0	lncRNA
110	*LNCARGI*	No	No	0	lncRNA
111	*LPP*	Yes	Yes, no.1	14	Protein-coding
112	*LRRC25*	Yes	Yes, no.4	3	Protein-coding
113	*LYST*	Yes	Yes, no.1	11	Protein-coding
114	*MAGI3*	Yes	Yes, no.8	2	Protein-coding
115	*MAPT-AS1*	Yes	No	0	Antisense
116	*MGAT5*	Yes	Yes, no.5	4	Protein-coding
117	*MICA-AS1*	No	No	0	Antisense
118	*MICB*	yes	Yes, no.4	5	Protein-coding
119	*MIR3142HG*	Yes	No	0	Mir_host

120	*MSRA*	Yes	Yes, no.3	6	Protein-coding
121	*MTCO3P1*	No	No	0	Pseudogene
122	*MUC21*	Yes	Yes, no.2	2	Protein-coding
123	*MYNN*	Yes	Yes, no.2	4	Protein-coding
124	*MYO16*	Yes	Yes, no.1	10	Protein-coding
125	*NAB1*	Yes	Yes, no.4	3	Protein-coding
126	*NAP1L4P1*	yes	No	0	Pseudogene
127	*NAPGP2*	No	No	0	Pseudogene
128	*NCF1*	Yes	No	5	Protein-coding
129	*NCF2*	Yes	Yes, no.2	9	Protein-coding
130	*NEMP2-DT*	No	No	0	Divergent transcript
131	*NEURL4*	Yes	Yes, no.1	5	Protein-coding
132	*NMNAT2*	Yes	Yes, no.2	5	Protein-coding
133	*NSF*	Yes	No	1	Protein-coding
134	*OVOL1*	Yes	Yes, no.2	6	Protein-coding
135	*PDHX*	Yes	Yes, no.1	8	Protein-coding
136	*PHRF1*	Yes	Yes, no.4	3	Protein-coding
137	*PHTF1*	Yes	Yes, no.1	8	Protein-coding
138	*PLCL1*	Yes	Yes, no.7	2	Protein-coding
139	*POLR1HASP*	Yes	Yes, no.3	1	Pseudogene
140	*POLR2LP1*	No	No	0	Pseudogene
141	*PRAG1*	Yes	No	2	Protein-coding
142	*PSORS1C1*	Yes	Yes, no.6	1	Protein-coding
143	*PTPN22*	Yes	Yes, no.1	7	Protein-coding
144	*PTPRC*	Yes	Yes, no.1	12	Protein-coding
145	*PXK*	Yes	Yes, no.1	10	Protein-coding
146	*RAD51B*	Yes	Yes, no.5	5	Protein-coding
147	*RASGRP1*	Yes	Yes, no.1	10	Protein-coding
148	*RASGRP3*	Yes	Yes, no.1	7	Protein-coding
149	*RGL1*	Yes	Yes, no.3	6	Protein-coding
150	*RMI2*	yes	Yes, no.2	4	Protein-coding

151	*RN7SKP62*	No	No	0	Pseudogene
152	*RNU6-376P*	Yes	No	0	Pseudogene
153	*RNU6-481P*	No	No	0	Pseudogene
154	*RNU6-682P*	No	No	0	Pseudogene
155	*RPL23AP91*	No	No	0	Pseudogene
156	*RPL3*	Yes	Yes, no.1	8	Protein-coding
157	*RSBN1*	Yes	Yes, no.1	6	Protein-coding
158	*SCAMP5*	Yes	Yes, no.9	2	Protein-coding
159	*SCGN*	Yes	Yes, no.7	2	Protein-coding
160	*SLC15A4*	Yes	Yes, no.1	7	Protein-coding
161	*SLC17A2*	Yes	Yes, no.5	4	Protein-coding
162	*SLC17A3*	Yes	Yes, no.2	3	Protein-coding
163	*SMG7*	Yes	Yes, no.1	4	Protein-coding
164	*SNHG32*	Yes	Yes, no.6	2	Protein-coding
165	*SNORA70*	Yes	Yes, no.1	2	Small nucleolar RNA
166	*SPMIP7*	Yes	Yes, no.2	2	Protein-coding
167	*SPPL3*	Yes	Yes, no.1	6	Protein-coding
168	*SPRED2*	Yes	Yes, no.1	5	Protein-coding
169	*SRGAP2*	Yes	Yes, no.1	9	Protein-coding
170	*SSBP4*	Yes	Yes, no.1	5	Protein-coding
171	*ST8SIA4*	Yes	Yes, no.5	4	Protein-coding
172	*STAT1*	Yes	Yes, no.4	17	Protein-coding
173	*STAT4*	Yes	Yes, no.2	5	Protein-coding
174	*SYNGR1*	Yes	Yes, no.1	4	Protein-coding
175	*TCF7*	Yes	Yes, no.3	3	Protein-coding
176	*TERT*	Yes	Yes, no.4	15	Protein-coding
177	*TIMMDC1*	Yes	Yes, no.2	3	Protein-coding
178	*TM9SF2*	Yes	Yes, no.1	5	Protein-coding
179	*TMEM39A*	Yes	Yes, no.5	3	Protein-coding
180	*TNFAIP3*	Yes	No	4	Protein-coding
181	*TNFSF4*	Yes	Yes, no.2	6	Protein-coding

182	*TNIP1*	Yes	Yes, no.1	5	Protein-coding
183	*TNPO3*	Yes	Yes, no.1	8	Protein-coding
184	*TNXB*	Yes	Yes, no.3	4	Protein-coding
185	*TPI1P2*	Yes	No	0	Pseudogene
186	*TSBP1*	Yes	Yes, no.1	4	Protein-coding
187	*TSBP1-AS1*	No	No	0	Antisense
188	*TYK2*	Yes	Yes, no.1	6	Protein-coding
189	*UBE2L3*	Yes	Yes, no.6	4	Protein-coding
190	*VDAC1*	Yes	Yes, no.1	18	Protein-coding
191	*WAKMAR2*	Yes	Yes, no.2	1	lncRNA
192	*WDFY4*	Yes	Yes, no.2	5	Protein-coding
193	*WNT3*	Yes	Yes, no.1	12	Protein-coding
194	*XKR6*	Yes	Yes, no.1	5	Protein-coding
195	es*Y_RNA*	Yes	No	0	Rho-associated RNA
196	*YDJC*	Yes	Yes, no.1	3	Protein-coding
197	*ZFP57*	Yes	Yes, no.3	3	Protein-coding
198	*ZNF76*	yes	Yes, no.1	4	Protein-coding

Supplementary table S2. *Selected set of BPA exposure studies for initial identification of BPA-sensitive CpG sites.*

**Study PMID**	**Study year**	**Study title**	**Experiment type**	**Study link**
31451752	2019	"An epigenome-wide analysis of cord blood DNA methylation reveals sex-specific effect of exposure to bisphenol A"	Environmental prenatal exposure; cord blood	https://www.ncbi.nlm.nih.gov/pmc/articles/PMC6710292/
34089743	2021	"Differential methylation of genes in the human placenta associated with bisphenol A exposure"	Environmental prenatal exposure; placenta	https://www.sciencedirect.com/science/article/pii/S0013935121006836
34200176	2021	"Prenatal Bisphenol a Exposure, DNA Methylation, and Low Birth Weight: A Pilot Study in Taiwan"	Environmental prenatal exposure; peripheral blood	https://pubmed.ncbi.nlm.nih.gov/34200176/
31601247	2019	"DNA methylome-wide alterations associated with estrogen receptor-dependent effects of bisphenols in breast cancer"	*In vitro* cell exposure; MCF7 cells	https://pubmed.ncbi.nlm.nih.gov/31601247/
37095195	2023	iGEM as a human iPS cell-based global epigenetic modulation detection assay provides throughput characterization of chemicals affecting DNA methylation	*In vitro* cell exposure; iGEM cells	https://pubmed.ncbi.nlm.nih.gov/37095195/
36217170	2022	Fetal exposure to phthalates and bisphenols and DNA methylation at birth: the Generation R Study	Environmental prenatal exposure; cord blood	https://pubmed.ncbi.nlm.nih.gov/36217170/
33391824	2020	Maternal environmental exposure to bisphenols and epigenome-wide DNA methylation in infant cord blood	Environmental prenatal exposure; cord blood	https://pubmed.ncbi.nlm.nih.gov/33391824/

Supplementary table S3. *CpG sites reported as differentially methylated according to authors criteria in ≥2 of the 7 selected BPA exposure studies (n=158 CpG sites). Chromosome positions are shown according to hg38 human genome assembly version.*

#chrom	*chromStart*	*chromEnd*	*name*
chr1	6128059	6128061	**cg12135344**
chr1	65254314	65254316	**cg11516629**
chr1	1.54E+08	153678822	**cg06444984**
chr10	1.01E+08	101131289	**cg02567119**
chr11	1.29E+08	128777213	**cg00344445**
chr14	53956206	53956208	**cg08162372**
chr14	70809199	70809201	**cg00025138**
chr15	40291507	40291509	**cg24111443**
chr16	11976448	11976450	**cg09401463**
chr16	51150650	51150652	**cg08526074**
chr16	85170028	85170030	**cg00942219**
chr17	4745966	4745968	**cg17900689**
chr17	16353659	16353661	**cg23401912**
chr19	589512	589514	**cg26260540**
chr19	2462066	2462068	**cg11879536**
chr2	37672211	37672213	**cg09973148**
chr22	19891652	19891654	**cg27306787**
chr5	88145263	88145265	**cg18344930**
chr6	31403229	31403231	**cg18204091**
chr6	1.7E+08	170244915	**cg11109139**
chr8	11201532	11201534	**cg02066409**
chr8	71843919	71843921	**cg09734791**
chr1	2E+08	200029342	**cg23996829**
chr13	83879144	83879146	**cg04095724**
chr14	92923530	92923532	**cg27018380**
chr16	86497991	86497993	**cg03731974**
chr17	63538551	63538553	**cg22097768**
chr3	1.8E+08	180037299	**cg18780412**
chr3	1.8E+08	180037448	**cg21176048**
chr6	30006541	30006543	**cg08879910**
chr6	32584261	32584263	**cg08578320**
chr6	72182893	72182895	**cg24401219**
chr1	18581603	18581605	**cg10574377**
chr1	25799968	25799970	**cg23931558**
chr1	32886406	32886408	**cg11273310**
chr1	40040109	40040111	**cg02998018**
chr1	54546734	54546736	**cg07576219**
chr1	77979219	77979221	**cg15821589**

chr1	98053544	98053546	**cg09896412**
chr1	1.13E+08	112957763	**cg19645639**
chr1	1.74E+08	173824799	**cg26937809**
chr1	2E+08	200029475	**cg18394216**
chr1	2.01E+08	201023529	**cg26228351**
chr10	43202692	43202694	**cg12451631**
chr10	73111319	73111321	**cg04833713**
chr10	1.01E+08	100997144	**cg25096861**
chr10	1.2E+08	120456867	**cg02150654**
chr11	4212271	4212273	**cg26730416**
chr11	9314033	9314035	**cg20070536**
chr11	15941385	15941387	**cg07805777**
chr11	58900486	58900488	**cg09527118**
chr11	64007598	64007600	**cg09182533**
chr11	74949219	74949221	**cg13987042**
chr11	1.19E+08	119423574	**cg13524082**
chr11	1.25E+08	124751934	**cg24942416**
chr12	189101	189103	**cg24087681**
chr12	11171411	11171413	**cg11506835**
chr12	24901970	24901972	**cg08980987**
chr12	49333326	49333328	**cg12760869**
chr12	1.14E+08	114409360	**cg06911121**
chr12	1.17E+08	116878686	**cg21334598**
chr12	1.31E+08	130714329	**cg27080194**
chr13	36430977	36430979	**cg06260815**
chr13	83878858	83878860	**cg18789918**
chr14	55440708	55440710	**cg10257302**
chr14	67619657	67619659	**cg13051728**
chr14	88324048	88324050	**cg05296192**
chr14	92923282	92923284	**cg21620282**
chr15	41494424	41494426	**cg01808706**
chr16	3046482	3046484	**cg05608716**
chr16	67029415	67029417	**cg09000178**
chr16	86496354	86496356	**cg04131583**
chr17	18153865	18153867	**cg10858337**
chr17	38720260	38720262	**cg18984002**
chr17	45034157	45034159	**cg20355401**
chr17	59619627	59619629	**cg02344993**
chr17	63538377	63538379	**cg22590258**
chr17	63961721	63961723	**cg17527484**
chr17	79799399	79799401	**cg03383056**
chr17	81035132	81035134	**cg14312959**
chr18	23134434	23134436	**cg06340447**
chr18	49561048	49561050	**cg17012181**
chr18	69401031	69401033	**cg23217126**
chr19	9435580	9435582	**cg07589201**

chr19	14689737	14689739	**cg00448895**
chr19	15551480	15551482	**cg13525067**
chr19	38224402	38224404	**cg20345042**
chr19	39498771	39498773	**cg25627144**
chr19	45178228	45178230	**cg09433379**
chr19	46493610	46493612	**cg13338783**
chr19	52297137	52297139	**cg24800326**
chr2	26786846	26786848	**cg24127278**
chr2	86195520	86195522	**cg20722537**
chr2	1.27E+08	127219038	**cg11107669**
chr2	1.61E+08	161415597	**cg09019329**
chr2	1.98E+08	197786157	**cg03774803**
chr2	2.24E+08	223602357	**cg20101489**
chr2	2.42E+08	241858895	**cg25890838**
chr20	24918353	24918355	**cg16892443**
chr20	58851802	58851804	**cg27262796**
chr20	62475975	62475977	**cg20265733**
chr20	62796335	62796337	**cg21431338**
chr20	63775367	63775369	**cg18369516**
chr21	32604611	32604613	**cg23545423**
chr22	50010931	50010933	**cg03900293**
chr3	18443918	18443920	**cg10742605**
chr3	27714108	27714110	**cg06088918**
chr3	44555353	44555355	**cg22147448**
chr3	52688435	52688437	**cg22692725**
chr3	1.09E+08	109316559	**cg01895333**
chr3	1.8E+08	180037232	**cg12864915**
chr4	5887926	5887928	**cg18417829**
chr4	26175475	26175477	**cg10937802**
chr4	39182203	39182205	**cg16968413**
chr4	41881154	41881156	**cg03905413**
chr4	84501299	84501301	**cg19441717**
chr4	1.54E+08	153789372	**cg05961809**
chr5	69093902	69093904	**cg18847612**
chr5	1.36E+08	135535877	**cg11144641**
chr6	24720196	24720198	**cg23113506**
chr6	26501752	26501754	**cg19729116**
chr6	27139938	27139940	**cg26190890**
chr6	27237545	27237547	**cg22982654**
chr6	28259613	28259615	**cg02578944**
chr6	28758011	28758013	**cg15416661**
chr6	29666717	29666719	**cg06301399**
chr6	29829207	29829209	**cg06316104**
chr6	31730445	31730447	**cg17983217**
chr6	32558249	32558251	**cg24638099**
chr6	37258172	37258174	**cg17307348**

chr6	44076587	44076589	**cg19930737**
chr6	72182596	72182598	**cg10034096**
chr6	89819787	89819789	**cg17390129**
chr6	1.06E+08	105981255	**cg00587009**
chr6	1.66E+08	165987531	**cg27088830**
chr6	1.69E+08	168884749	**cg20619067**
chr7	5425175	5425177	**cg08021273**
chr7	26858065	26858067	**cg03730533**
chr7	27169650	27169652	**cg05250768**
chr7	31052197	31052199	**cg20165074**
chr7	79454128	79454130	**cg04652097**
chr7	1.01E+08	101238895	**cg22188918**
chr7	1.22E+08	121872909	**cg10115498**
chr7	1.58E+08	157685155	**cg04207084**
chr8	10335419	10335421	**cg05753693**
chr8	17577031	17577033	**cg16800975**
chr8	23163579	23163581	**cg10964421**
chr8	23709796	23709798	**cg12812583**
chr8	85245163	85245165	**cg20503956**
chr8	92095377	92095379	**cg13111243**
chr8	1.19E+08	119417279	**cg19197744**
chr8	1.44E+08	143716167	**cg22043634**
chr8	1.45E+08	144529467	**cg23712522**
chr9	38069323	38069325	**cg05037166**
chr9	99823386	99823388	**cg14569576**
chr9	1.37E+08	136985772	**cg14348741**
chrX	56232261	56232263	**cg19505129**
chrY	19076585	19076587	**cg03244189**

Supplementary table S4. *Selected BPA-sensitive CpG sites, methylation effects in original studies and DepMap, annotated genes and interactions according to CTD.*

CpG name	BPA methylation effects: original studies (OS), DepMap (DM) †	Gene (distance to TSS, bp) ‡	BPA-gene interactions in CTD (n) ¶	Chemical-gene interactions in CTD (n) ¶
cg12135344	Down/up (OS), **down (DM)**	*CHD5* (+52,063)	2	18
		KCNAB2 (+82,139)	3	28
cg11516629	**Up (OS)**, down (DM)	*DNAJC6* (-55,230)	1	14
		AK4 (+106,485)	2	18
cg06444984	**Up (OS)**, down (DM)	*NPR1* (+184)	1	9
cg02567119	Down/up (OS), **up (DM)**	*TLX1* (+783)	2	18
cg00344445*	**Down (OS)**, down (DM)	*KCNJ1* (+65,322)	0	7
		FLI1 (+83,442)	4	28
cg08162372	**Down (OS)**, down (DM)	*BMP4* (+604)	0	11
cg00025138	Down/up (OS), **down (DM)**	*MAP3K9* (+334)	1	6
cg08526074	Down/up (OS), **up (DM)**	*SALL1* (+624)	3	19
cg00942219	Down/up (OS), **down (DM)**	*GSE1* (-443,187)	2	24
		FAM92B (-57,521)	No data	No data
cg17900689*	**Down (OS)**, down (DM)	*TM4SF5* (-25,925)	0	8
		ZMYND15 (+5,952)	0	8
cg23401912	Down/up (OS), **down (DM)**	*CENPV* (-161)	1	5
cg26260540	**Up (OS)**, down (DM)	*HCN2* (-380)	1	12
cg11879536	**Down (OS)**, up (DM)	*GADD45B* (-14,060)	0	10
		LMNB2 (-5,071)	0	5
cg09973148	**Down (OS)**, up (DM)	*CDC42EP3* (-21)	3	10
cg27306787	Down/up (OS), **down (DM)**	*COMT* (-49,954)	1	108
		GNB1L (-36,714)	0	11
cg18344930	Down/up (OS), **down (DM)**	*CCNH* (-732,245)	0	1
		TMEM161B (+123,566)	0	6
cg18204091*	Down/up (OS), **down (DM)**	*MICB* (-94,885)	2	15
		HLA-B (-46,043)	0	6
cg11109139	**Up (OS)**, down (DM)	*DLL1* (+45,559)	0	13
		ERMARD (+493,289)	0	4
cg09734791	Down/up (OS), **down (DM)**	*MSC* (+548)	3	11

*SLE-colocalized CpG sites defined as CpG sites located within 50 kilobase pairs from SLE single nucleotide variants (SNVs) or with GREAT-annotated genes (https://great.stanford.edu/great/public/html/index.php, default settings) overlapping with GWAS catalogue SLE genes.

†Annotated genes defined as CpG sites located within 50 kilobase pairs from SLE single nucleotide variants (SNVs) or with GREAT-annotated genes overlapping with GWAS catalogue SLE genes. Negative Z-score values, i.e. Z-score values from hypomethylated BPA-sensitive CpG sites, were multiplied by -1 if differentially methylated in the same direction as in the seven human BPA studies. If the results from the original studies were inconclusive ("Down/up"), methylation direction in annotated genes according to DepMap data was used. The methylation directions of effect in the original studies or DepMap used for calculation of the BPA scores are marked in bold with "down" indicating hypomethylation and "up" indicating hypermethylation. TSS, transcription start site; bp, base pairs.

¶ Number of annotated Bisphenol A – gene interactions for methylation compared to total number of annotated Chemical – gene interactions for methylation reported in the Comparative toxicogenomic database (CTD, https://ctdbase.org/).

Supplementary figure S1. *Distribution of the BPA_{All} score across patients and controls in the discovery nd replication cohort. Differences in the BPA_{All} score between patients and controls were assessed using a two-sided Mann-Whitney U test with $p<0.05$ defining significance. Discovery, discovery cohort; Replication, replication cohort.*

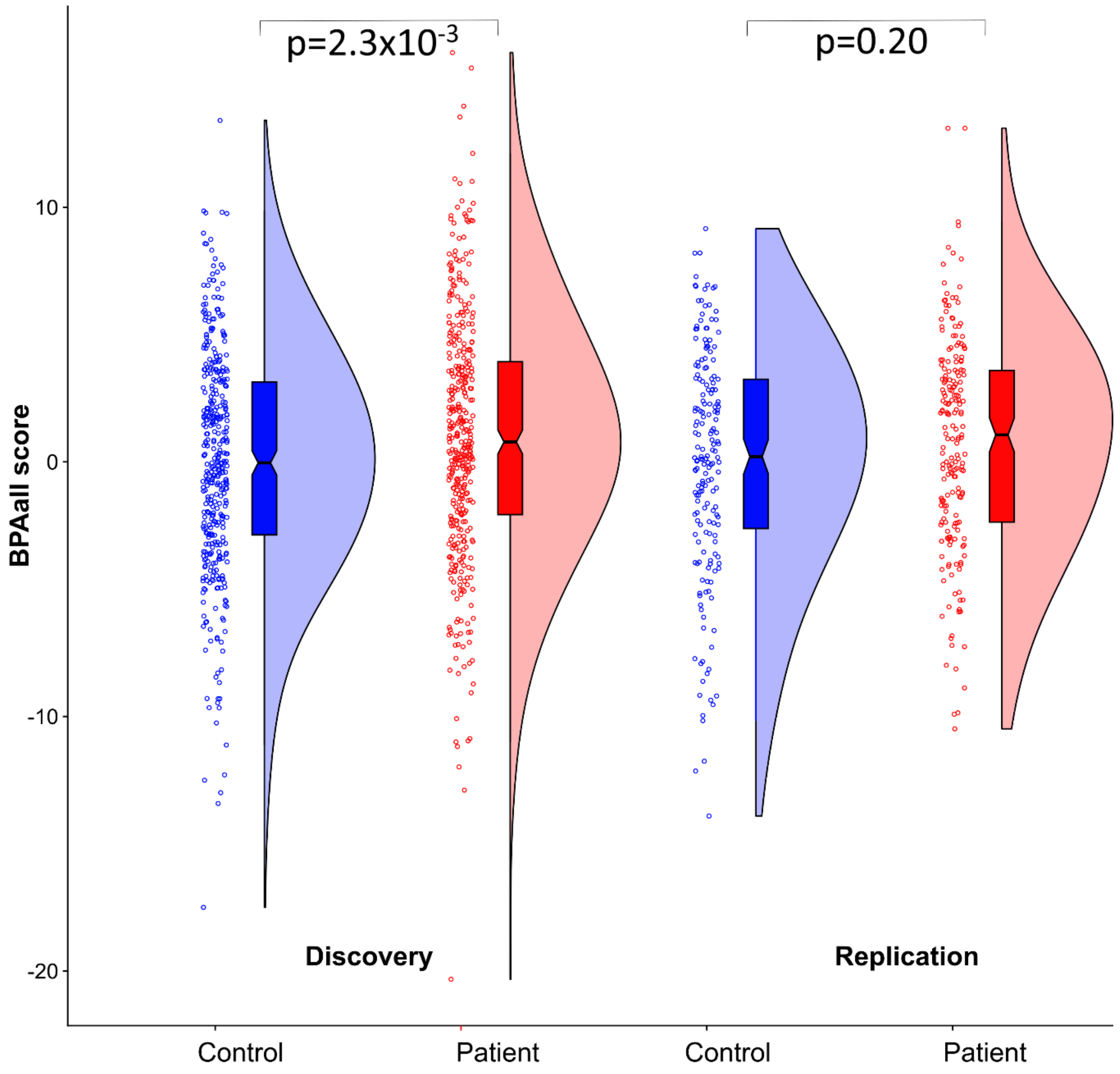

Supplementary figure S2. Number of chemical-gene interactions for methylation reported in CTD for *MICB* and *FLI1*. Unfiltered output.

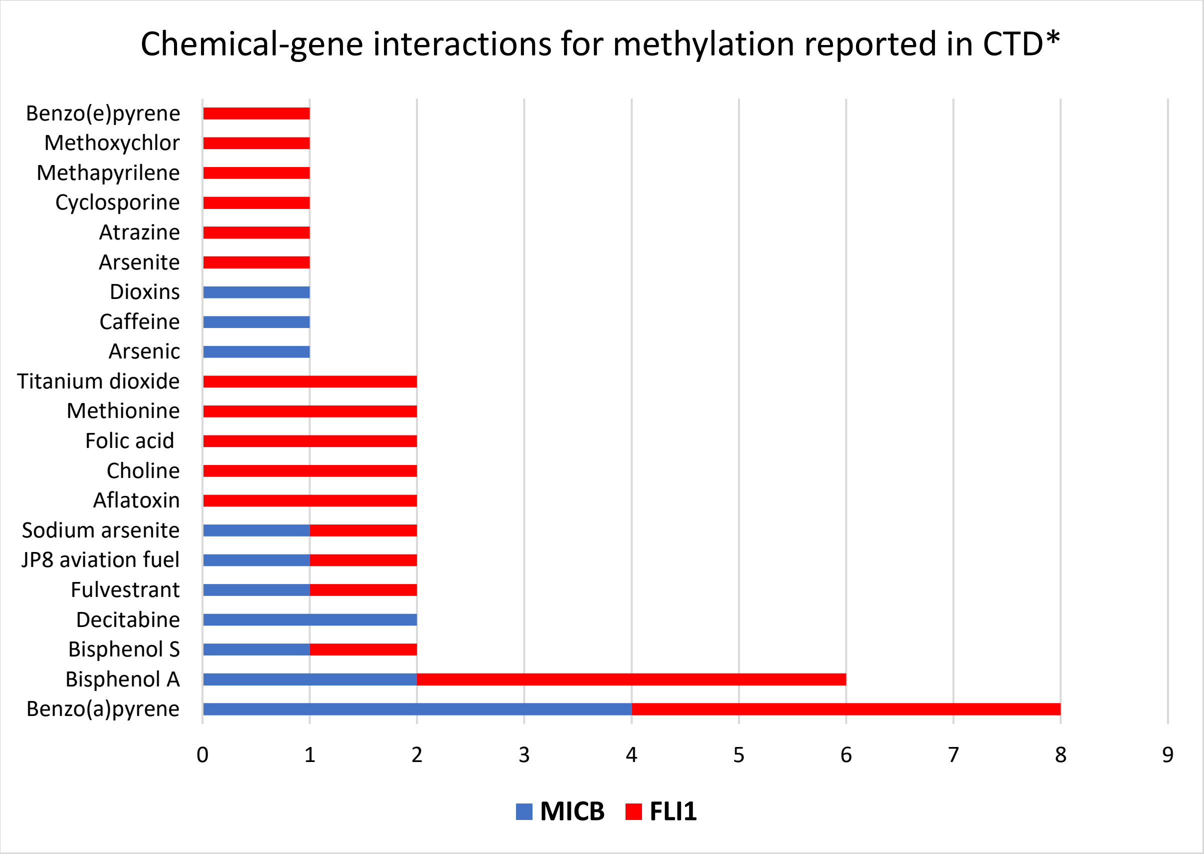

***CTD:** Comparative Toxicogenomics Database (https://ctdbase.org/)

Supplementary table S5. *Basic characteristics of the cohorts.*

	Discovery			Replication			
	**Patients**	**Controls**	**p-value***	**Patients**	**Controls**	**p-value***	**p-value †**
**N total**	347	400		201	187		
**Mean age at sampling (±S.D.)**	47.0 (17.2)	47.1 (13.2)	0.72	48.7 (14.8)	48.7 (14.9)	0.95	0.18
**Female (%)**	300 (86.5)	351 (87.8)	0.60	175 (87.1)	174 (93.0)	0.050	0.84
**Male (%)**	47 (13.5)	49 (12.3)	0.60	26 (12.9)	13 (7.0)	0.050	0.84
**Mean BPA_{All} score (±S.D.)**	1.07 (5.00)	0.00 (4.39)	$2.6x10^{-3}$	0.76 (4.20)	0.00 (4.50)	0.20	0.46
**Mean BPA_{SLE} score (±S.D.)**	1.12 (2.10)	0.00 (1.80)	$3.5x10^{-14}$	0.98 (1.99)	0.01 (1.92)	$1.1x10^{-5}$	0.45
**Mean age at diagnosis (±S.D.)**	34.7 (17.0)			34.7 (14.5)			0.50
**Mean disease duration (±S.D.)**	12.3 (11.4)			14.0 (12.3)			0.14
**Mean total ACR-82 criteria (±S.D.) ‡**	5.24 (1.21)			5.67 (1.35)			
**Mean SLEDAI (n=343) ‡**	3.06 (4.16)						
**Mean SLAM (n=200) ‡**				6.56 (4.17)			

*Independent samples T-test, Mann-Whitney U test or Pearson Chi-square test. Analysis of differences between patients and controls in the discovery and replication cohort respectively.

†Independent samples T-test, Mann Whitney U-test or Pearson Chi-Square Test. Analysis of differences between patients from the discovery cohort and patients from the replication cohort.

‡ACR-82 criteria, American College of Rheumatology-82 criteria; SLEDAI, Systemic Lupus Erythematosus Disease Activity Index [2]; SLAM, Systemic Lupus Activity Measure [3].

Supplementary table S6. *Clinical data of the two separate cohorts including American College of Rheumatology-82 (ACR-82) criteria, autoantibodies and medication.*

	**Discovery cohort N (%)**	**Replication cohort N (%)**	**P-value***
**ACR1 Malar rash**	188 (54.2)	103 (51.2)	0.51
**ACR2 Discoid rash**	76 (21.9)	34 (16.9)	0.16
**ACR3 Photosensitivity**	203 (58.5)	134 (66.7)	0.058
**ACR4 Oral ulcers**	66 (19.0)	49 (24.4)	0.14
**ACR5 Arthritis**	254 (73.2)	166 (82.6)	0.012
**ACR6 Serositis**	145 (41.8)	82 (40.8)	0.82
**ACR7 Renal**	103 (29.7)	77 (38.3)	**0.038**
**ACR8 Neurologic**	20 (5.8)	21 (10.4)	**0.045**
**ARC9 Hematologic**	211 (60.8)	140 (69.7)	**0.038**
**ACR10 Immunologic**	211 (60.8)	136 (67.7)	0.11
**ACR11 ANA**	340 (98.0)	198 (98.5)	0.66
**SSA**	153 (45.1)	87 (43.3)	0.68
**SSB**	91 (26.8)	50 (24.9)	0.63
**Anti-RNP**	114 (33.5)	56 (27.9)	0.17
**Anti-Sm**	45 (13.2)	29 (14.4)	0.70
**Anti-dsDNA**	200 (58.8)	127 (63.2)	0.32
**Prednisolone**	215 (62.0)	111 (55.5)	0.14
**Antimalarials**	166 (47.8)	73 (60.8)	**0.014**
**Azathioprine**	55 (15.9)	37 (24.3)	**0.024**
**Mycophenolate**	36 (10.4)	18 (10.2)	0.96
**Methotrexate**	22 (6.3)	8 (4.8)	0.47
**DMARD**	113 (32.6)	60 (42.0)	**0.048**

*Pearson Chi-Square test. Analysis of differences in clinical status between cohorts.

Supplementary table S7. *Associations between the BPA_{All} score and clinical subphenotypes.*

		**BPA_{All}**	
	**N (%)**	**OR (95% CI)**	**p**
**Sex (female)**	475 (86.7)	0.97 (0.92-1.03)	0.34
**Age at sampling ≥60**	145 (26.5)	0.96 (0.92-1.00)	0.051
**ACR-criteria***			
**Malar rash**	291 (53.1)	0.99 (0.96-1.03)	0.69
**Discoid lupus**	110 (20.1)	0.98 (0.94-1.03)	0.48
**Photosensitivity**	337 (61.5)	0.98 (0.94-1.01)	0.22
**Oral ulcers**	115 (21.0)	1.01 (0.97-1.06)	0.54
**Arthritis**	420 (76.6)	1.03 (0.99-1.07)	0.17
**Serositis**	227 (41.4)	1.02 (0.99-1.06)	0.22
**Glomerulonephritis**	180 (32.8)	1.00 (0.96-1.04)	1.00
**Neurologic disorder**	41 (7.5)	1.05 (0.98-1.12)	0.16
**Hematologic disorder**	351 (64.1)	0.99 (0.95-1.03)	0.54
**Immunologic disorder**	347 (63.3)	1.01 (0.97-1.05)	0.65
**Antinuclear antibodies**	538 (98.2)	0.97 (0.85-1.11)	0.69
**Anti-SSA**	240 (44.4)	0.99 (0.96-1.03)	0.78
**Anti-SSB**	141 (26.1)	1.01 (0.97-1.05)	0.76
**Anti-RNP**	170 (31.4)	1.03 (0.99-1.07)	0.094
**Anti-Sm**	74 (13.7)	1.01 (0.96-1.06)	0.72
**Anti-dsDNA**	327 (60.4)	1.01 (0.97-1.05)	0.62
**Sum ab ≥3†**	62 (11.5)	1.01 (0.97-1.06)	0.52
**SLAM>6 or SLEDAI >4‡**	154 (28.4)	0.99 (0.96-1.03)	0.77

Simple logistic regression. Unadjusted p-values are presented with p<0.05 marked in bold.

*Disease manifestations according to the American College of Rheumatology 82-criteria for SLE (ACR-82).

†Sum anti-SSA, anti-SSB, anti-RNP, anti-Sm and anti-dsDNA ≥3. ‡High disease activity defined as SLE activity measure (SLAM) >6 or SLE disease activity index (SLEDAI) >4 [2, 3].

Supplementary figure S3. *Functional enrichment for genes shown to be differentially expressed in at least two of the four cell lines. Length of lines indicate fold enrichment; dot size corresponds to number of genes and color indicates the negative logarithm with base 10 of the false discovery rate (-log10(FDR)). Plot shown based on ShinyGO enrichment service (https://bioinformatics.sdstate.edu/go/).*

A. *Plot of top 25 significant PANTHER pathways.*

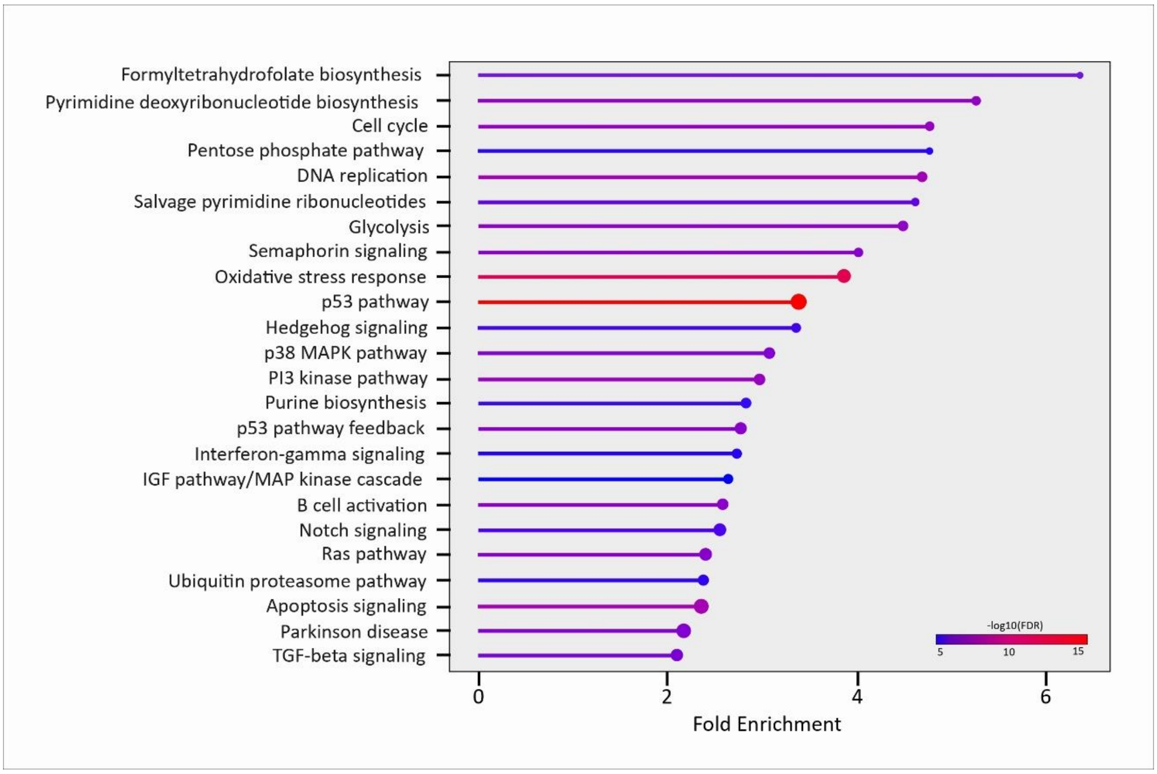

B. *Plot of top 20 significant Hallmark signatures.*

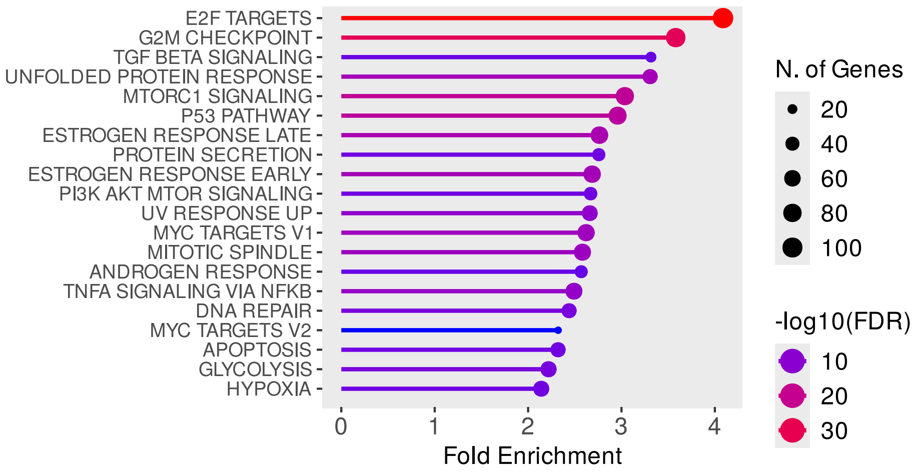

Supplementary figure S4. *Enrichment plot with selected top Hallmark signatures according to MSigDB for the differentially expressed genes in the four BPA-treated cell lines.*

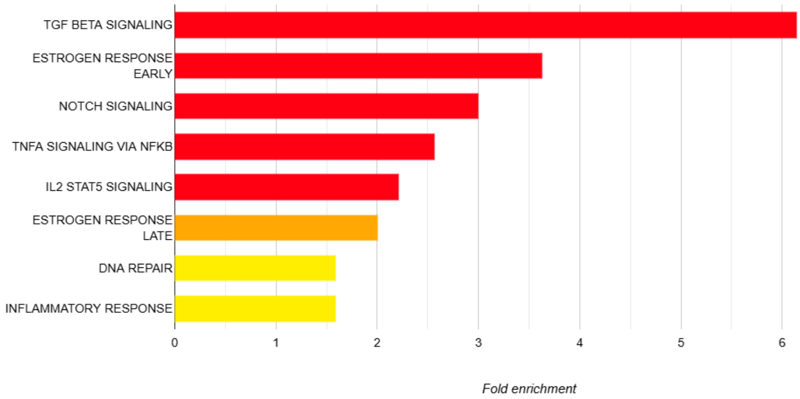

MCF7

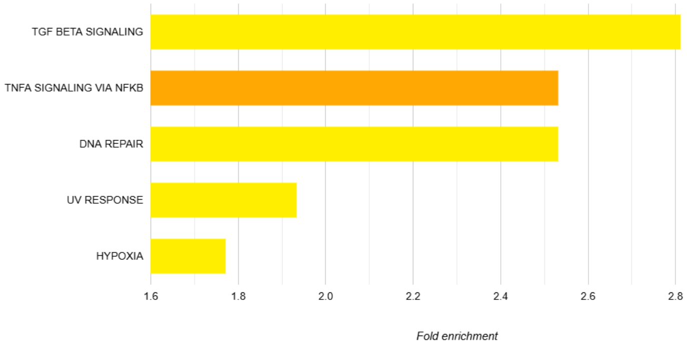

HepG2

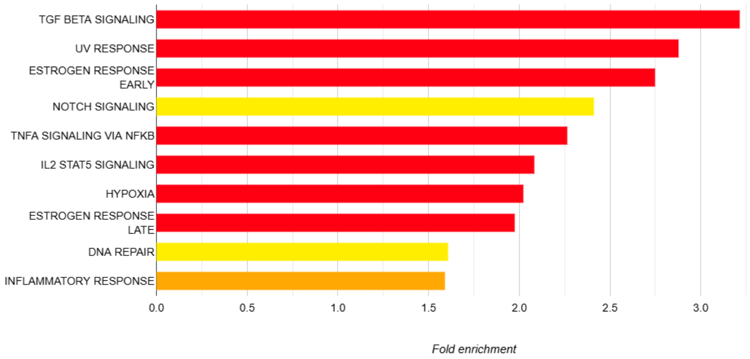

Ishikawa

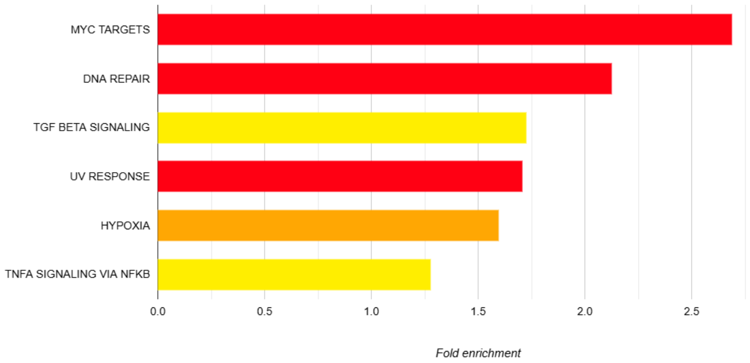

Y79

Supplementary figure S5. *Functional enrichment analysis of genes, differentially expressed in BPA-treated lymphoblastoid cells. Top 100 Reactome pathways shown with. Number of genes shown. Immune-relevant pathways highlighted in red.*

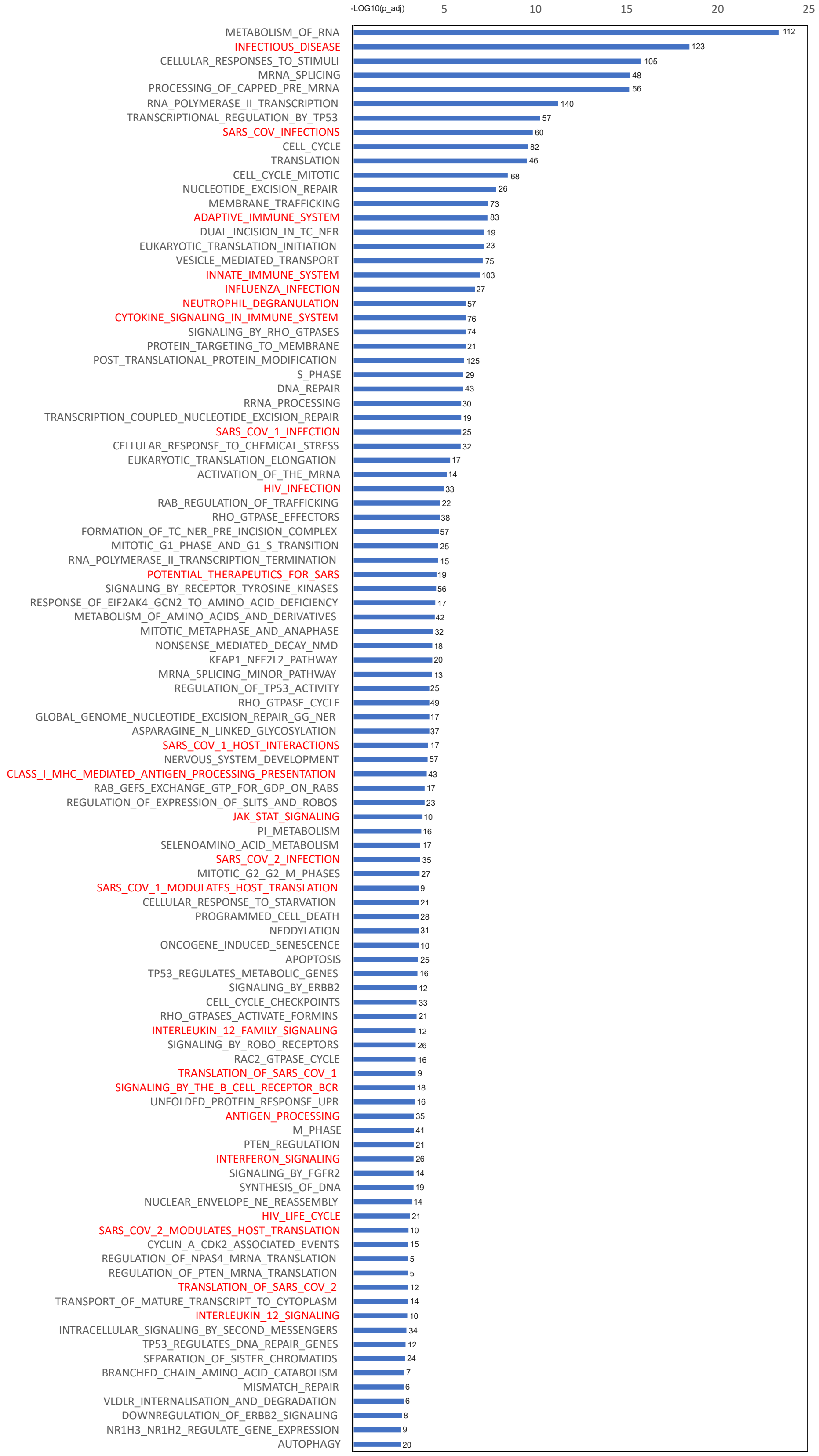

Supplementary table S8. *BPA-associated CpG annotated genes and interferon regulation according to Interferome with the settings any treatment concentrations, any treatment time, only humans, in vitro and in vivo, all organs, all cells, all cell lines and any sample types.*

CpG	Annotated gene(s)	Interferon-regulated	Interferon type		
			I	II	III
**cg12135344**	*CHD5* (+52,063)				
	KCNAB2 (+82,139)	Yes	Yes	Yes	
**cg11516629**	*DNAJC6* (-55,230)	Yes		Yes	
	AK4 (+106,485)	Yes	Yes	Yes	
**cg06444984**	*NPR1* (+184)	Yes	Yes		
**cg02567119**	*TLX1* (+783)				
**cg00344445***	*KCNJ1* (+65,322)	Yes		Yes	
	FLI1 (+83,442)	Yes		Yes	
**cg08162372**	*BMP4* (+604)	Yes		Yes	
**cg00025138**	*MAP3K9* (+334)	Yes	Yes		
**cg08526074**	*SALL1* (+624)	Yes		Yes	
**cg00942219**	*GSE1* (-443,187)	Yes	Yes	Yes	
	FAM92B (-57,521)				
**cg17900689***	*TM4SF5* (-25,925)				
	ZMYND15 (+5,952)	Yes	Yes	Yes	
**cg23401912**	*CENPV* (-161)	Yes		Yes	
**cg26260540**	*HCN2* (-380)	Yes		Yes	Yes
**cg11879536**	*GADD45B* (-14,060)	Yes	Yes	Yes	
	LMNB2 (-5,071)	Yes		Yes	
**cg09973148**	*CDC42EP3* (-21)	Yes	Yes	Yes	
**cg27306787**	*COMT* (-49,954)				
	GNB1L (-36,714)	Yes		Yes	
**cg18344930**	*CCNH* (-732,245)	Yes		Yes	
	TMEM161B (+123,566)	Yes	Yes		
**cg18204091***	*MICB* (-94,885)	Yes	Yes	Yes	
	HLA-B (-46,043)	Yes	Yes	Yes	
**cg11109139**	*DLL1* (+45,559)	Yes	Yes	Yes	
	ERMARD (+493,289)				
**cg09734791**	*MSC* (+548)	Yes	Yes	Yes	

*SLE-colocalized CpG sites defined as CpG sites located within 50 kilobase pairs from SLE single nucleotide variants (SNVs) or with GREAT-annotated genes (https://great.stanford.edu/great/public/html/index.php, default settings) overlapping with GWAS catalogue SLE genes.

Supplementary table S9. *Differential expression of genes, based on BPA treatment of the four cell lines and of the set of BPA-treated lymphoblastoid cell, that were also found to be among the genes neighboring BPA-sensitive CpG sites.*

**Type**	**Cell source (GEO dataset ID)**	**Gene**	**Expression changes**	**logFC**	**Adjusted p-value**
Cancer cell line	Ishikawa (GSE69849)	*GADD45B*	Up	0.17	5.5x10^{-3}
		COMT	Down	-0.13	1.1x10^{-2}
		MICB	Up	0.24	9.4x10^{-6}
	HepG2 (GSE69850)	*TMEM161B*	Up	0.14	3.2x10^{-2}
	Y79 (GSE146255)	*GADD45B*	Up	0.74	7.6x10^{-3}
		TMEM161B	Up	0.7	3.3x10^{-4}
		CENPV	Down	-0.67	1.4x10^{-6}
		DNAJC6	Up	0.59	2.3x10^{-3}
		HCN2	Up	0.65	3.7x10^{-3}
		MAP3K9	Up	1.17	2.1x10^{-17}
		SALL1	Down	-0.31	1.3x10^{-2}
	MCF7 (GSE271332)	*GADD45B*	Down	-1.94	1.7x10^{-3}
		COMT	Down	-0.98	3.5x10^{-5}
		LMNB2	Up	0.66	4.8x10-^{2}
EBV-transformed B-cells	Lymphoblastoid cells (GSE207049)	*CCNH*	Up	0.29	5.5x10-^{4}
		GADD45B	Up	0.25	2.1x10-^{2}
		GSE1	Up	0.26	1.4x10-^{2}
		HLA-B	Up	0.18	7.3x10-^{3}
		KCNAB2	Up	0.25	8.0x10-^{3}
		MAP3K9	Down	-0.42	3.9x10-^{2}
		MICB	Down	-0.44	2.5x10-^{3}
		TMEM161B	Down	-0.41	7.1x10-^{4}

References

1. Davis AP, Wiegers TC, Sciaky D, Barkalow F, Strong M, Wyatt B, et al. Comparative Toxicogenomics Database's 20th anniversary: update 2025. Nucleic Acids Res. 2025;53(D1):D1328-d34.

2. Gladman DD, Ibañez D, Urowitz MB. Systemic lupus erythematosus disease activity index 2000. J Rheumatol. 2002;29(2):288-91.

3. Liang MH, Socher SA, Roberts WN, Esdaile JM. Measurement of systemic lupus erythematosus activity in clinical research. Arthritis & Rheumatism. 1988;31(7):817-25.
